# Comparative analysis of *Lactobacillus gasseri* from Chinese subjects reveals a new species-level taxa

**DOI:** 10.1186/s12864-020-6527-y

**Published:** 2020-02-03

**Authors:** Xingya Zhou, Bo Yang, Catherine Stanton, R. Paul Ross, Jianxin Zhao, Hao Zhang, Wei Chen

**Affiliations:** 1 State Key Laboratory of Food Science and Technology, Jiangnan University, Wuxi, Jiangsu China; 2 School of Food Science and Technology, Jiangnan University, 1800 Lihu Avenue, Wuxi, 214122 China; 3 International Joint Research Center for Probiotics & Gut Health, Jiangnan University, Wuxi, Jiangsu China; 4 Teagasc Food Research Centre, Moorepark, Fermoy, Cork, Ireland; 5 APC Microbiome Ireland, University College Cork, Cork, Ireland; 6 National Engineering Research Center for Functional Food, Jiangnan University, Wuxi, Jiangsu China; 7 Wuxi Translational Medicine Research Center and Jiangsu Translational Medicine Research Institute Wuxi Branch, Wuxi, China; 8 Beijing Innovation Center of Food Nutrition and Human Health, Beijing Technology and Business University (BTBU), Beijing, China

**Keywords:** *Lactobacillus paragasseri*, *Lactobacillus gasseri*, Pan/core-genome, ANI, Genotype

## Abstract

**Background:**

*Lactobacillus gasseri* as a probiotic has history of safe consumption is prevalent in infants and adults gut microbiota to maintain gut homeostasis.

**Results:**

In this study, to explore the genomic diversity and mine potential probiotic characteristics of *L. gasseri*, 92 strains of *L. gasseri* were isolated from Chinese human feces and identified based on 16 s rDNA sequencing, after draft genomes sequencing, further average nucleotide identity (ANI) value and phylogenetic analysis reclassified them as *L. paragasseri* (*n* = 79) and *L. gasseri* (*n* = 13), respectively. Their pan/core-genomes were determined, revealing that *L. paragasseri* had an open pan-genome. Comparative analysis was carried out to identify genetic features, and the results indicated that 39 strains of *L. paragasseri* harboured Type II-A CRISPR-Cas system while 12 strains of *L. gasseri* contained Type I-E and II-A CRISPR-Cas systems. Bacteriocin operons and the number of carbohydrate-active enzymes were significantly different between the two species.

**Conclusions:**

This is the first time to study pan/core-genome of *L. gasseri* and *L. paragasseri*, and compare their genetic diversity, and all the results provided better understating on genetics of the two species.

## Background

*Lactobacillus gasseri*, as one of the autochthonous microorganism colonizes the oral cavity, gastrointestinal tract and vagina of humans, has a variety of probiotic properties [1]. Clinical trials indicated that *L. gasseri* maintains gut and vaginal homeostasis, mitigates *Helicobacter pylori* infection [2] and inhibits some virus infection [3], which involve multifaceted mechanisms such as production of lactic acid, bacteriocin and hydrogen peroxide [4], degradation of oxalate [5], protection of epithelium invasion by pathogens exclusion [6].

Initially, it was difficult to distinguish *L. gasseri*, *Lactobacillus acidophilus* and *Lactobacillus johnsonii*, and later *L. gasseri* was reclassified as a separate species by DNA-DNA hybridization techniques [7], 16S rDNA sequencing [8] and repetitive element-PCR (Rep-PCR) [9] from the close related species. Sequencing technologies and whole-genome-based analysis made the clarification of taxonomical adjunct species more accurate [10, 11]. Nevertheless, no further investigation was performed on its subspecies or other adjunct species in recent years. ANI values were considered as a useful approach to evaluate the genetic distance, based on genomes [12, 13]. The ANI values were higher than 62% within a genus, while more than 95% of ANI values was recommended as the delimitation criterion for same species [14]. Seventy-five *L. gasseri* strains with publically available genomes were divided into two intraspecific groups by ANI at the threshold of 94% [15], subsequently some strains were re-classified as a new group, *L. paragasseri*, based on whole-genome analysis [16].

Sequencing technologies and bioinformatics analysis provide the opportunities to analyse more information of microbial species. Pan-genome is a collection of multiple genomes, including core genome and variable genome. The core genome consists of genes presented in all strains and is generally associated with biological functions and major phenotypic characteristics, reflecting the stability of the species. And variable genome consists of genes that exist only in a single strain or a portion of strains, and is generally related to adaptation to particular environments or to unique biological characteristics, reflecting the characteristics of the species [17]. Pan-genomes of other *Lactobacillus* species [18], such as *Lactobacillus reuteri* [19], *Lactobacillus paracasei* [20], *Lactobacillus casei* [21] and *Lactobacillus salivarius* [22] have previously been characterized. The genetic knowledge and diversity of *L. gasseri* and *L*. *paragasseri* is still in its infancy. In addition, previous in silico surveys have reported that *Lactobacilli* harbour diverse and active CRISPR-Cas systems, which has 6-fold-rate occurrence of CRISPR-Cas systems compared with other bacteria [23]. It is necessary to study CRISPR-Cas system to understand the adaptive immune system that protect *Lactobacillus* from phages and other invasive mobile genetic elements in engineering food microbes, and explore powerful genome engineering tool. Moreover, numerous bacteriocins were isolated from *Lactobacillus* genus, and these antimicrobials received increased attention as potential alternatives to inhibit spoilage and pathogenic bacteria [24]. A variety of strategies identify bacteriocin culture-based and in silico-based approaches, and to date, bacteriocin screening by in silico-based approaches have been reported in many research investigations [25].

In the current work, strains were isolated from fecal samples collected from different regions in China, and initially identified as *L. gasseri* by 16S rDNA sequencing. For further investigation, draft genomes of all the strains were sequenced by next generation sequencing (NGS) platform and analysed by bioinformatics to explore the genetic diversity, including subspecies/adjunct species, pan-genome, CRISPR-Cas systems, bacteriocin and carbohydrate utilization enzymes.

## Results

### Strains and sequencing

Based on 16S rDNA sequencing, 92 *L. gasseri* strains were isolated from fecal samples obtained from adults and children from different regions in China, with 66 strains being obtained from adults and 26 from children (47 strains were isolated from females, 45 were isolated from males) (Table 1). The draft genomes of all strains were sequenced using Next Generation Sequencing (NGS) technology and strains were sequenced to a coverage depth no less than the genome 100 ×, and using the genome of *L. gasseri* ATCC33323 and *L. paragasseri* K7 as reference sequences.

**Table 1:** General features of eight complete genomes of *L. paragasseri* and *L. gasseri*

Strain	Host (Age range)	Gender	Region	Size (Mb)	GC (%)	tRNA	Genes	ORF	Hypothetical proteins	Reference
*L. gasseri* ATCC33323	–	–	Japan	1.89	35.3	75	1891	1754	–	[26]
*L. paragasseri* K7	–	–	Slovenia	1.99	34.8	55	1991	1826	–	[27]
FAHFY1-L2	0–1	Female	Fuyang	1.92	34.7	51	1842	1893	18.17%	This work
FAHFY7-L4	0–1	Female	Fuyang	1.89	34.75	51	1928	1814	14.06%	This work
FBJHD4-L7	0–1	Male	Beijin	1.93	34.67	48	1904	1838	13.82%	This work
FFJFZ1-L2	0–1	Female	Fuzhou	1.96	34.77	51	1893	1888	17.62%	This work
FFJND16-L4	0–1	Male	Ningde	1.86	34.8	53	2025	1858	17.33%	This work
FFJND2-L7	0–1	Male	Ningde	1.97	34.94	52	1887	1987	19.28%	This work
FFJND4-L5	0–1	Female	Ningde	1.94	34.72	49	1887	1858	12.81%	This work
FFJND5-L1	0–1	Female	Ningde	1.97	34.93	52	2019	1976	18.62%	This work
FFJND6-L1	0–1	Female	Ningde	1.95	34.71	51	1878	1841	13.09%	This work
FFJND7-L1	0–1)	Female	Ningde	1.95	34.74	45	1874	1838	12.95%	This work
FGSYC10-L1	40–50	Female	Yongchang	2	35.5	56	1980	1988	13.63%	This work
FGSYC15-L1	70–80	Male	Yongchang	1.98	35.3	56	2006	2022	19.98%	This work
FGSYC18-L5	60–70	Female	Yongchang	1.96	34.9	47	1878	1934	15.05%	This work
FGSYC19-L1	60–70	Female	Yongchang	1.95	35.3	52	1955	1990	20.05%	This work
FGSYC23-L3	60–70	Male	Yongchang	1.95	34.86	39	1880	1942	15.40%	This work
FGSYC2-L2	60–70	Male	Yongchang	1.96	34.92	37	1884	1949	15.70%	This work
FGSYC34-L2	50–60	Female	Yongchang	1.95	34.87	40	1877	1939	15.37%	This work
FGSYC38-L3	50–60	Male	Yongchang	1.96	34.78	52	1853	1894	14.68%	This work
FGSYC41-L1	60–70	Male	Yongchang	1.97	35.4	56	1947	1954	15.51%	This work
FGSYC43-L1	50–60	Female	Yongchang	1.98	34.76	48	1921	1970	16.40%	This work
FGSYC79-L2	10–20	Male	Yongchang	1.99	34.93	54	1948	1987	16.46%	This work
FGSYC7-L1	50–70	Male	Yongchang	1.91	34.71	37	1827	1853	13.65%	This work
FGSYC8-L2	60–70	Male	Yongchang	2.01	34.84	35	1972	2030	19.46%	This work
FGSYC9-L1	70–80	Female	Yongchang	1.95	35.3	53	1910	1906	14.64%	This work
FGSZY12-L1	10–20	Male	Zhangye	1.94	34.82	41	1850	1878	13.79%	This work
FGSZY27-L1	1–10	Male	Zhangye	1.95	35.4	42	1881	1880	11.49%	This work
FGSZY29-L8	1–10	Male	Zhangye	2.01	35.3	53	2004	2003	13.98%	This work
FGSZY30-L1	1–10	Male	Zhangye	1.99	35.3	53	1980	1977	13.66%	This work
FGSZY36-L1	1–10	Male	Zhangye	1.99	35.3	53	1973	1974	13.68%	This work
FHeBCZ3-L3	0–1	Female	Cangzhou	2.02	34.76	49	2029	1958	16.96%	This work
FHeNJZ11-L9	0–1	Female	Jiaozuo	1.91	34.72	51	1879	1795	9.64%	This work
FHLJDQ3-L5	20–30	Male	Daqing	1.92	34.74	52	1916	1902	18.45%	This work
FHNFQ10-L1	50–60	Male	Fengqiu	2.04	34.66	40	1993	2005	15.21%	This work
FHNFQ11-L7	70–80	Male	Fengqiu	2.05	35.2	60	2033	2018	13.23%	This work
FHNFQ14-L5	60–70	Male	Fengqiu	2.11	35.4	56	2137	2157	15.67%	This work
FHNFQ15-L4	60–70	Female	Fengqiu	2.14	35.4	56	2169	2206	16.00%	This work
FHNFQ16-L5	60–70	Female	Fengqiu	1.99	35.3	55	1993	1999	18.41%	This work
FHNFQ20-L1	70–80	Female	Fengqiu	2.08	35.3	58	2051	2040	15.83%	This work
FHNFQ25-L3	50–60	Female	Fengqiu	1.88	34.62	40	1816	1852	17.28%	This work
FHNFQ28-L4	0–1	Female	Fengqiu	2.01	35.3	53	1956	1940	15.41%	This work
FHNFQ29-L2	50–60	Female	Fengqiu	2.02	35.3	57	1964	1974	14.34%	This work
FHNFQ34-L1	70–80	Female	Fengqiu	2.09	34.82	41	1875	2166	16.67%	This work
FHNFQ3-L8	20–30	Female	Fengqiu	1.9	35.2	54	2089	1885	17.19%	This work
FHNFQ46-L1	60–70	Male	Fengqiu	1.87	34.63	37	1860	1893	17.33%	This work
FHNFQ53-L2	60–70	Female	Fengqiu	2	35.3	54	2017	2057	20.86%	This work
FHNFQ56-L1	60–70	Female	Fengqiu	1.94	34.89	52	1909	1950	19.03%	This work
FHNFQ57-L4	40–50	Male	Fengqiu	1.88	35.3	54	1797	1796	8.46%	This work
FHNFQ60-L1	80–90	Male	Fengqiu	1.89	34.68	47	1817	1865	17.59%	This work
FHNFQ62-L6	60–70	Female	Fengqiu	1.96	34.74	44	1866	1909	13.93%	This work
FHNFQ63-L6	60–70	Female	Fengqiu	1.96	34.74	42	1863	1906	13.96%	This work
FHNXY12-L2	60–70	Male	Xiayi	1.99	34.86	53	1932	1983	16.44%	This work
FHNXY18-L2	60–70	Female	Xiayi	1.93	34.62	46	1921	1987	19.78%	This work
FHNXY26-L3	0–1	Female	Xiayi	1.92	34.66	50	1889	1926	18.38%	This work
FHNXY28-L4	10–20	Male	Xiayi	2.04	34.81	43	2055	2087	17.01%	This work
FHNXY29-L1	60–70	Male	Xiayi	1.9	34.78	44	1817	1869	13.48%	This work
FHNXY34-L1	60–70	Male	Xiayi	1.94	34.76	47	1799	1834	13.85%	This work
FHNXY44-L1	80–90	Female	Xiayi	1.98	34.76	42	1911	1953	15.98%	This work
FHNXY46-L6	80–90	Female	Xiayi	1.94	34.73	39	1827	1862	13.75%	This work
FHNXY49-L5	80–90	Female	Xiayi	1.95	34.76	40	1902	1942	14.06%	This work
FHNXY52-L2	50–60	Female	Xiayi	1.92	34.63	36	1822	1854	13.48%	This work
FHNXY54-L2	50–60	Female	Xiayi	2	34.77	38	1965	2012	15.95%	This work
FHNXY56-L1	50–60	Female	Xiayi	1.93	34.75	47	1915	1961	17.80%	This work
FHNXY58-L2	80–90	Female	Xiayi	1.87	34.78	43	1914	1842	17.92%	This work
FHNXY61-L1	90–100	Male	Xiayi	2.01	34.77	41	1958	2016	15.38%	This work
FHNXY6-L2	40–50	Male	Xiayi	2	34.81	52	1804	2003	16.33%	This work
FHNXY9-L1	60–70	Male	Xiayi	1.97	35	48	1914	1973	19.31%	This work
FHuNCS1-L1	1–10	Male	Changsha	1.96	34.72	41	1983	1952	19.31%	This work
FJSCZD2-L1	60–70	Male	Changzhou	1.97	34.83	53	2057	1972	18.20%	This work
FJSSZ1-L1	0–1	Female	Suzhou	1.88	34.63	48	1907	1843	11.01%	This work
FJSWX10-L4	0–1	Female	Wuxi	1.91	34.7	48	1934	1871	17.10%	This work
FJSWX21-L2	0–1	Male	Wuxi	1.99	34.81	55	1950	1913	16.62%	This work
FJSWX33-L2	0–1	Female	Wuxi	2.04	34.74	52	2053	1984	17.24%	This work
FJSWX6-L7	0–1	Female	Wuxi	2.01	34.89	47	2012	1963	18.34%	This work
FJSWX9-L2	0–1	Male	Poyang	1.9	34.67	55	1935	1874	18.36%	This work
FJXPY18-L3	70–80	Male	Poyang	1.92	34.95	68	1907	1851	13.83%	This work
FJXPY24-L2	50–60	Female	Poyang	2.08	34.81	37	2102	2033	17.41%	This work
FJXPY26-L4	10–20	Male	Poyang	1.98	34.68	48	2023	1989	18.65%	This work
FJXPY34-L1	50–60	Female	Poyang	1.94	34.71	36	1921	1881	18.77%	This work
FJXPY37-L3	50–60	Male	Poyang	1.9	34.72	57	1968	1870	11.71%	This work
FJXPY5-L2	50–60	Female	Poyang	1.98	34.78	55	1989	1885	11.67%	This work
FJXPY6-L1	10–20	Male	Poyang	1.98	34.8	73	1986	1892	11.68%	This work
FNMGHHHT1-L5	0–1	Female	Huhhot	1.95	34.68	37	1987	1932	19.31%	This work
FNMGHLBE17-L3	1–10	Male	Hulunbuir	2	34.69	48	1962	1903	17.76%	This work
FNMGHLBE20-L5	40–50	Female	Hulunbuir	2	34.69	45	1947	1924	15.64%	This work
FNMGHLBE6-L1	20–30	Female	Hulunbuir	1.94	34.61	46	1890	1849	17.31%	This work
FSDHZ19-L1	0–1	Male	Heze	1.9	34.83	25	1888	1830	12.08%	This work
FSDHZ21-L1	0–1	Male	Heze	1.95	34.89	40	1947	1897	16.97%	This work
FSDHZD3-L5	20–30	Female	Heze	1.98	34.96	56	2069	2016	20.34%	This work
FSDYT1-L1	0–1	Male	Yantai	2.02	34.86	55	1952	1932	16.10%	This work
FTJWQ2-L9	0–1	Male	Tianjin	1.96	34.86	53	1882	1828	12.09%	This work
FZJHZD1-M5	60–70	Female	Hangzhou	1.96	34.92	53	1991	1934	16.65%	This work
M2CF21-L1	30–40	Male	Tibet	1.94	34.68	47	1897	1846	13.49%	This work

“-”: unknown

### ANI values

**Fig. 1:**
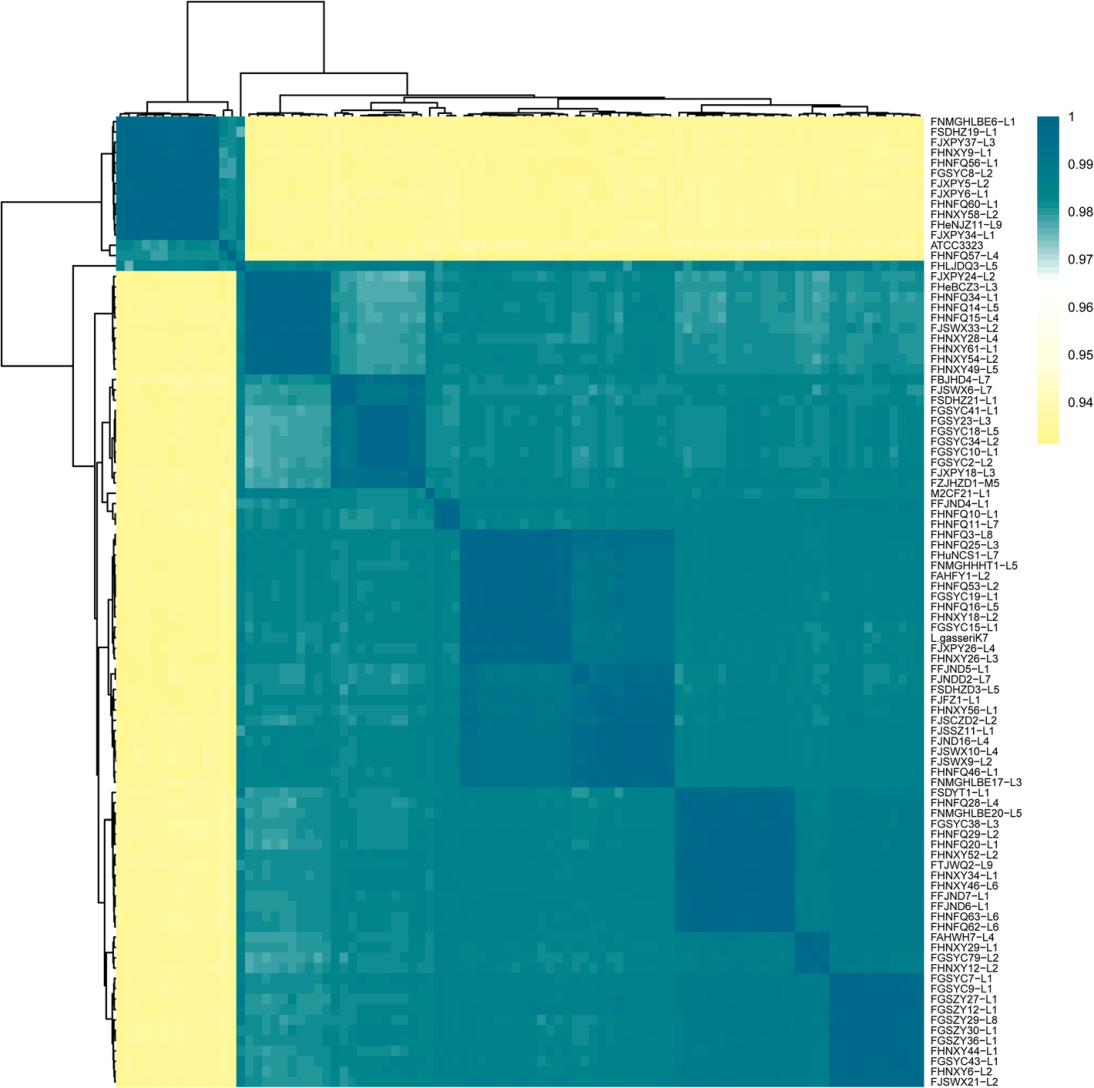
Average nucleotide identity (ANI) alignment of all the strains including *L. gasseri* ATCC33323 and *L. paragasseri* K7

ANI values calculation of Z92 draft genomes was carried out through pairwise comparison at the 95% threshold to further identify their species (Fig. 1). All of the 94 strains were classified into two groups, with 80 strains including *L. paragasseri* K7 (as type *L. paragasseri* strain) showing an ANI value range 97–99%, and the other group consisted of 14 strains including the type strain *L. gasseri* ATCC 33323 (as type *L. gasseri* strain) with an ANI range 93–94% compared with *L. paragasseri*. According to a previous report, *L. gasseri* K7 was reclassified as *L. paragasseri* based on whole-genome analyses [16], therefore, other 79 strains on the same group with *L. paragasseri* K7 were preliminarily identified as *L. paragasseri*, while the remained 13 strains on the other branch with *L. gasseri* ATCC33323 were identified as *L. gasseri*.

### Phylogenetic analysis

**Fig. 2:**
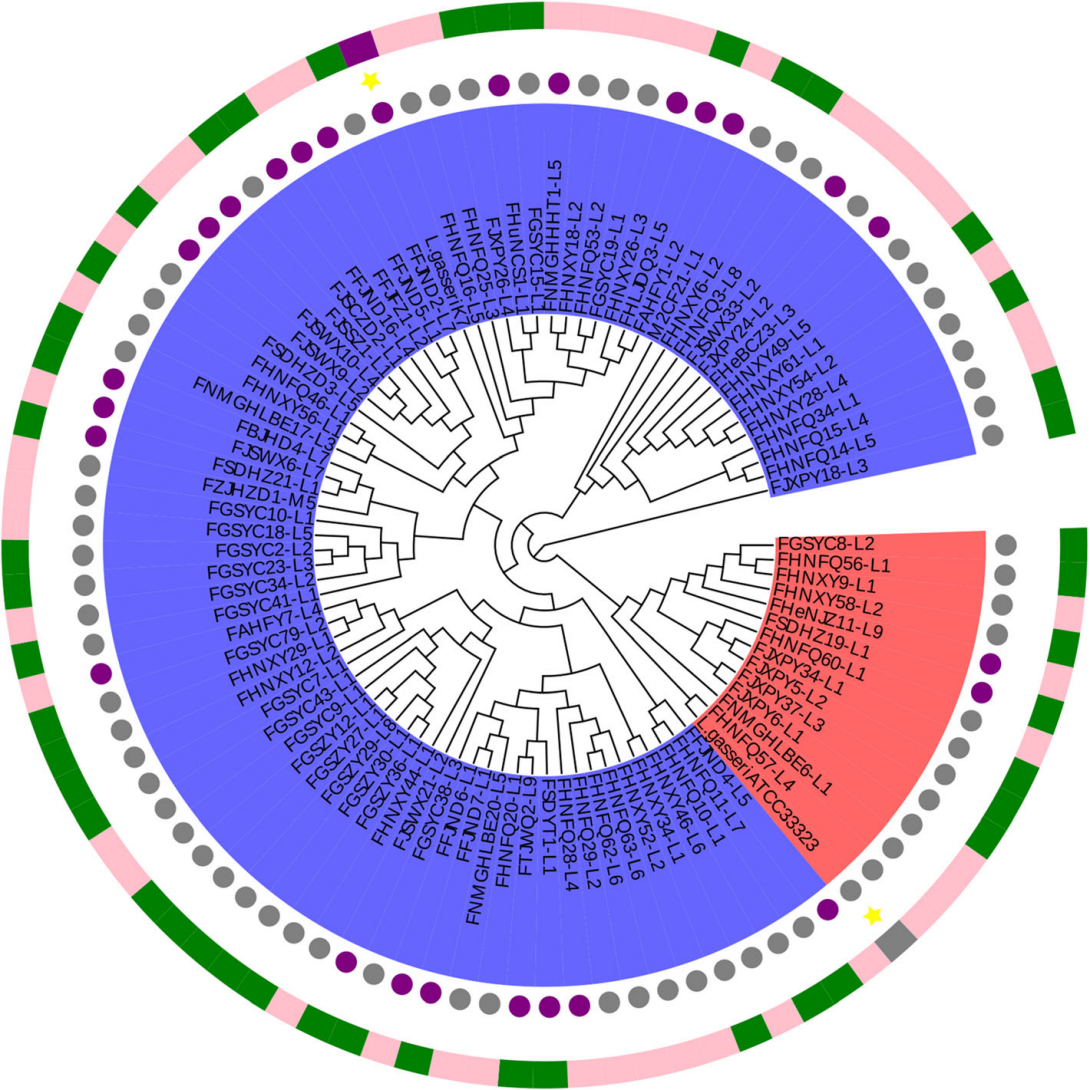
The phylogenetic tree based on orthologous genes. The red area was the *L. gasseri* cluster and the blue area was the *L. paragasseri* cluster. The purple circle indicated the strains isolated from infant feces and the gray indicated strains isolated from adults. The pink indicated strains from female and the green represent strains from male subjects

**Fig. 3:**
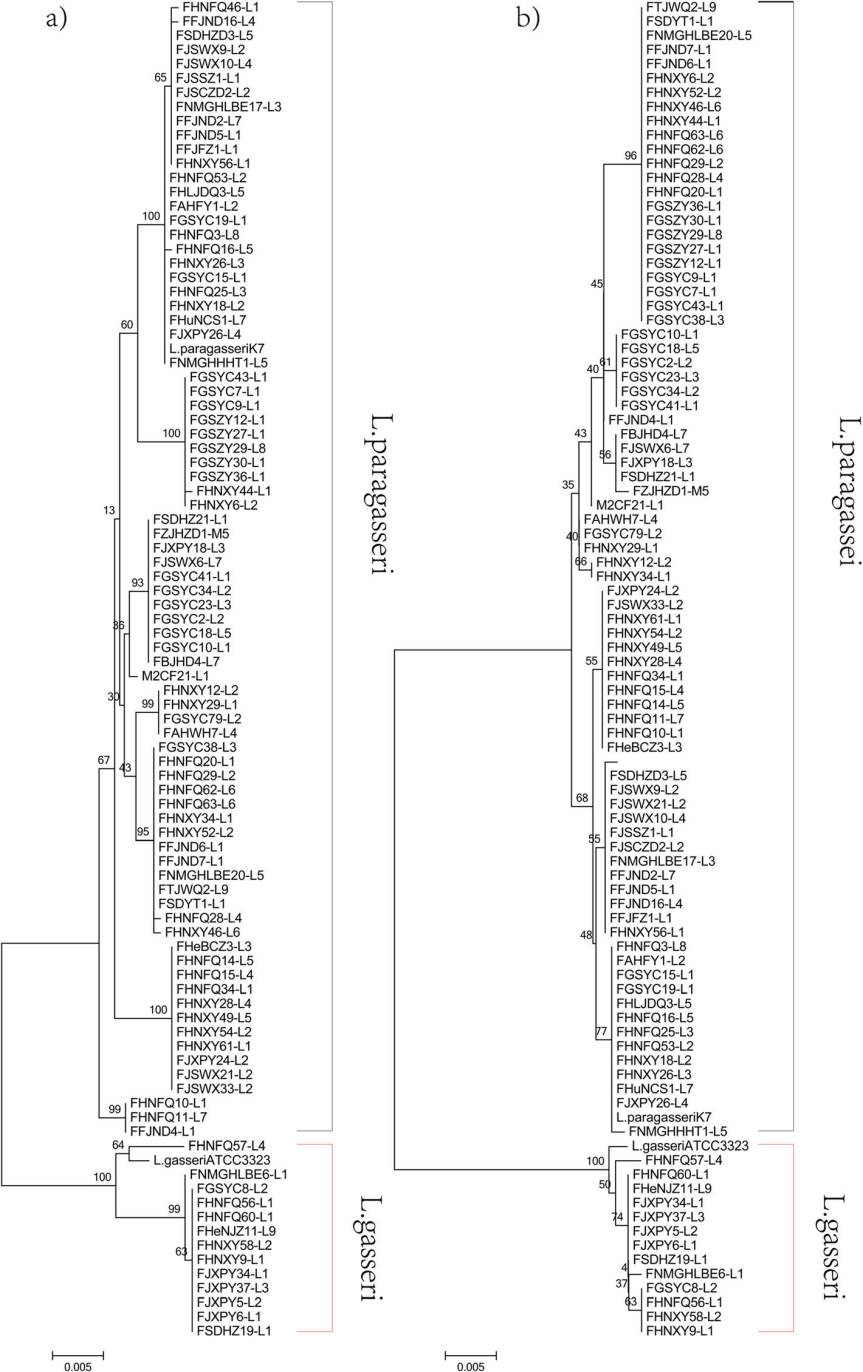
Neighbor-joining tree based on *groEL* (**a**) and *pheS* (**b**) gene

To further verify the results from ANI and evaluate the genetic distance among strains, the phylogenetic relationships between *L. paragasseri* and *L. gasseri* were investigated. OrthoMCL was used to cluster orthologous genes and 1282 orthologues proteins were shared by all the 94 genomes. A robust phylogenetic tree based on 1282 orthologues proteins was constructed (Fig. 2). The results indicated that all the 94 strains could be positioned on two branches, in which 80 strains were on the same cluster with *L. paragasseri* K7 and the other 14 strains were on the cluster with *L. gasseri* ATCC33323. Surprisingly, all the strains on the cluster with *L. gasseri* or *L. paragasseri* were completely consistent with the results from ANI analysis. Therefore, it was confirmed that division of 92 strains isolated from Chinese subjects into two subgroups; 79 strains belong to *L. paragasseri*, and 13 strains to *L. gasseri*, is correct. The strains were randomly selected from the fecal samples, suggesting that *L. gasseri* and *L. paragasseri* had no preference to either male or female subjects nor region and age. Moreover, the house-keeping genes *pheS* and *groEL* were extracted from the genomes and neighbor-joining trees were built. The tree showed that 13 strains of *L. gasseri* were clustered in a single clade (Fig. 3), which was consistent with phylogenetic data based on orthologous genes. However, there were many branches in the *L. paragasseri* groups*,* which indicated a high intraspecies diversity among *L. paragasseri* and needs further investigation (Fig. 2, Fig. 3).

### General genome features and annotation

The general information of the 80 genomes of *L. paragasseri* strains and 14 genomes of *L .gasseri* strains are summarized in Table 1. The sequence length of *L. paragasseri* ranged from 1.87 to 2.14 Mb, with a mean size of 1.97 Mb, and all 14 *L. gasseri* genomes had an average sequence length of 1.94 Mb with a range of 1.87–2.01 Mb. The *L. paragasseri* genomes displayed an average G + C content of 34.9% and *L. gasseri* genomes had an average G + C content of 34.82%. A comparable number of predicted Open Reading Frames (ORF) was obtained for each *L. paragasseri* genome that ranged from 1814 to 2206 with an average number of 1942 ORFs per genome, while *L. gasseri* had an average number of 1881 ORFs per genome. To further determine the function of each gene, non-redundant protein databases based on NCBI database were created, which revealed that average 84% of *L. paragasseri* ORFs were identified, while the remaining 16% were predicted to encode hypothetical proteins. Similarly, approximately 85% of *L. gasseri* ORFs were identified, while 15% were predicted to encode hypothetical proteins. The preference of the two species codons for the start codon were predicted, and the results showed that ATG, TTG and CTG in *L. paragasseri* with a calculated frequency percentage of 82.6, 10.3 and 7.1%, respectively, and 81.0, 11.7 and 7.4% in *L. gasseri*, respectively, suggesting that *L. paragasseri* and *L. gasseri* had a preference of using ATG as start codon [16].

To further analyse the genome-encoded functional proteins, the COG classification was performed for each draft genome. According to the results of the COG annotation, the genes were divided into 20 groups, and the details are shown in (Additional file 1: Table S1) and (Additional file 2: Table S2). The results revealed that carbohydrate transport and metabolism, defense mechanisms differed in different genomes of *L. paragasseri*, while *L. gasseri* showed only difference in defense mechanisms. Notably, due to draft genomes, the possibility of error from missing genes or incorrect copy number is significantly higher [28].

### Pan/core-genome analysis

**Fig. 4:**
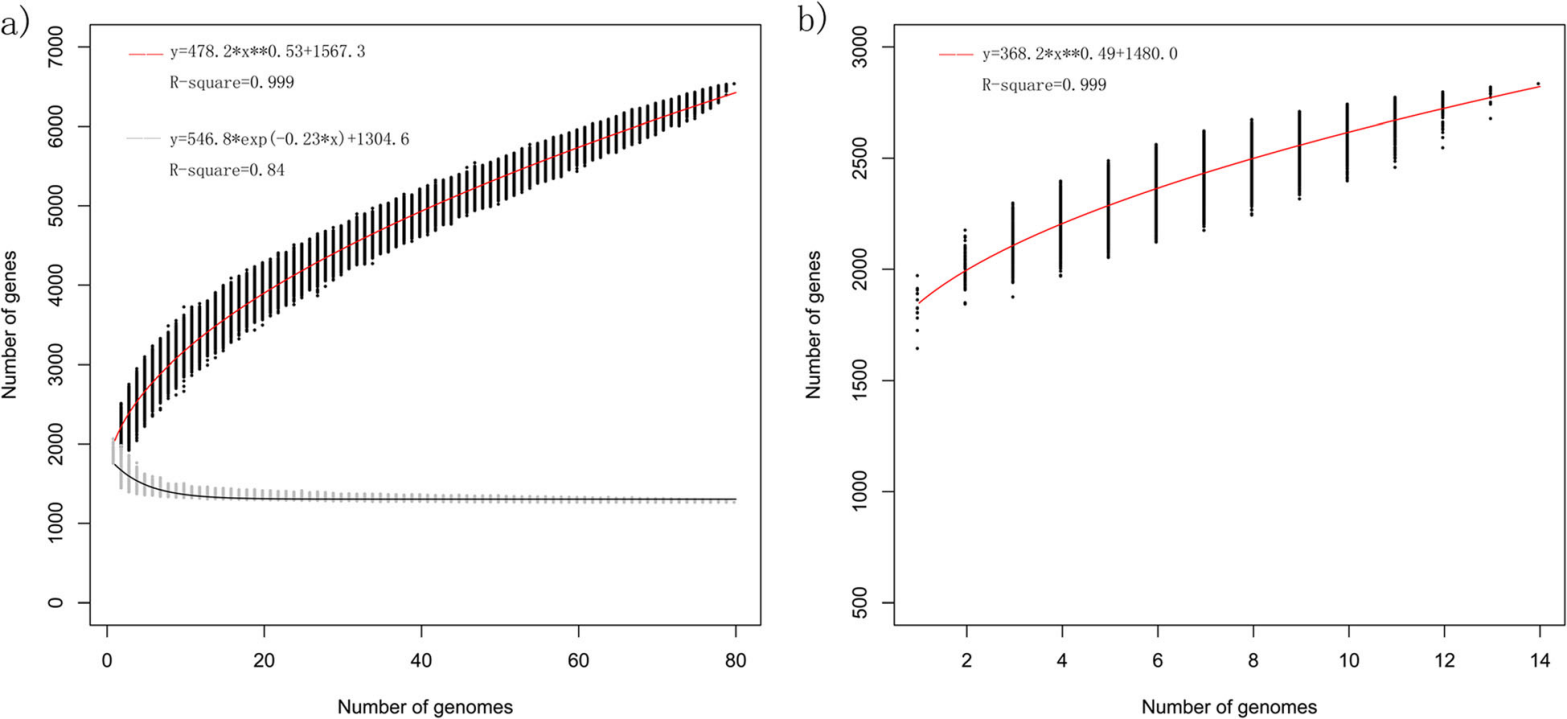
Pan-genome and core-genome curve of the *L. paragasseri* (**a**) and *L. gasseri* (**b**)

To analyze the overall approximation of the gene repertoire for *L. paragasseri* and *L. gasseri* in the human intestine, the pan-genomes of *L. paragasseri* and *L. gasseri* were investigated, respectively. The results showed that the pan-genome size of all 80 strains of *L. paragasseri* amounted to 6535 genes while the pan-genome asymptotic curve had not reached a plateau (Fig. 4), suggesting that when more *L. paragasseri* genomes were considered for the number of novel genes, the pan-genome would continuously increase. Meanwhile, the exponential value of deduced mathematical function is > 0.5 (Fig. 4), these findings indicated an open pan-genome occurrence within the *L. paragasseri* species. *L. paragasseri* had a supragenome about 3.3 times larger than the average genome of each strain, indicating *L. paragasseri* constantly acquired new genes to adapt to the environment during evolution. The pan-genome size of the 14 strains of *L. gasseri* was 2834 genes, and the exponential value of deduced mathematical function is < 0.5, thus it could not be concluded whether its pan-genome was open or not.

**Fig. 5:**
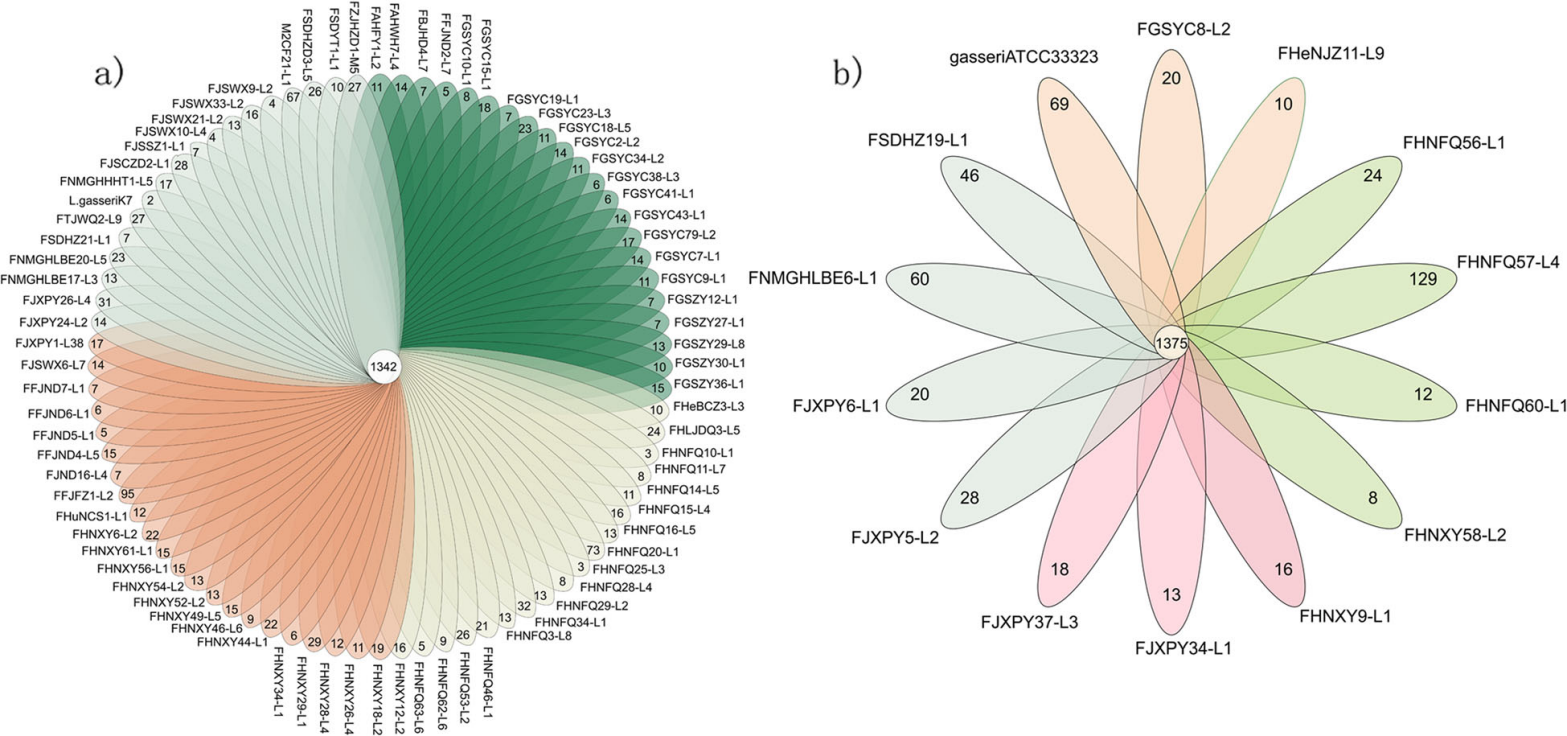
The unique and orthologues genes of *L. paragasseri* genomes (**a**) and *L. gasseri* (**b**)

The number of conserved gene families constituting the core genome decreased slightly, and the extrapolation of the curve indicated that the core genome reached a minimum of 1256 genes in *L. paragasseri* and 1375 genes in *L. gasseri*, and the curve of *L. paragasseri* remained relatively constant, even as more genomes were added. The Venn diagram represented the unique and orthologues genes among the 80 *L. paragasseri* strains*.* The unique orthologous clusters ranged from 3 to 95 genes for *L. paragasseri* and ranged from 8 to 125 genes for *L. gasseri* (Fig. 5). As expected, the core genome included a large number of genes for translation, ribosomal structure, biogenesis and carbohydrate transport and metabolism, in addition to a large number of genes with unknown function (Additional file 5: Figure S1).

### Identification and characterization of CRISPR in *L. paragasseri* and *L. gasseri*

The CRISPR-Cas adaptive immunity system provided resistance against invasive bacteriophage or plasmid DNA such as some lytic bacteriophages in engineering food microbes, which consists of CRISPR adjacent to *Cas* genes. The presence of Cas1 proteins was used to determine the presence or absence of CRISPR-Cas systems, and Cas1 was found among the 39 strains of *L. paragasseri*, and 13 strains of *L. gasseri*. The occurrence of *Cas1* genes in *L. paragasseri* and *L. gasseri* showed differences, in that 12 strains of *L. gasseri* consisted of two *Cas1* genes, and the second *Cas1* gene was located in a different region constituting a second putative CRISPR locus. Meanwhile, *Cas2* and *Cas9* were widespread across the two species, while *Cas3*, *Cas5*, *Cas6* and *Cas7* only occurred in *L. gasseri*. According to previous method of classification of the CRISPR subtypes, 52 Type II-A systems were detected in all the strains of *L. gasseri* and 39 strains of *L. paragasseri*, whereas the Type I-E system only occurred in 12 strains of *L. gasseri* except FHNFQ57-L4, indicating that subtype II-A was the most prevalence both in *L. paragasseri* and *L. gasseri.*

**Fig. 6:**
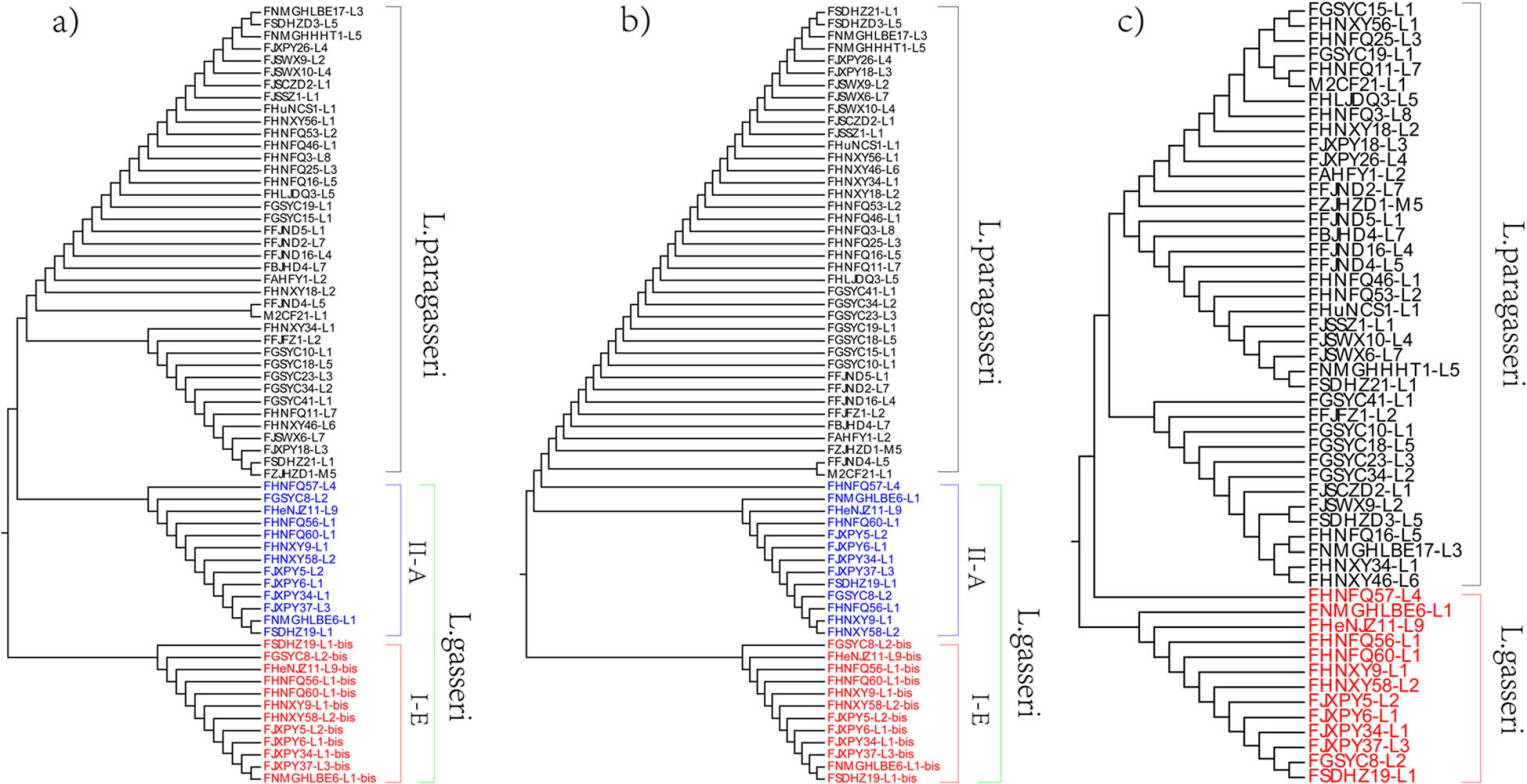
CRISPR-cas phylogenetic analyses for *L. paragasseri* and *L. gasseri*. **a** Phylogenetic tree based on the Ca1 protein, **b** Phylogenetic tree based on the Cas2 protein, **c** Phylogenetic tree based on the Cas9 protein. The CRISPR-Cas subtypes and bacterial species were written on the right and each group was colored

The phylogenetic analyses performed with *Cas1*, *Cas2* and *Cas9* from the two species showed *L. paragasseri* was clearly distinct from *L. gasseri* (Fig. 6). Strikingly, phylogenetic tree based on Cas1 and Cas2 proteins revealed that clusters consisted of only the second Cas1 and Cas2 proteins in Type I-E systems in *L. gasseri*, and the Cas1 and Cas2 proteins in subtype II-A systems in both *L. paragasseri* and *L. gasseri* were clustered in two groups. From this perspective, CRISPR-Cas could be used as an indicator to distinguish *L. paragasseri* and *L. gasseri*. Moreover, phylogenetic analysis of Cas9 indicated that the cluster was consistent with *Cas1* and *Cas2*, indicating that co-evolutionary trends happened in CRISPR systems.

**Fig. 7:**
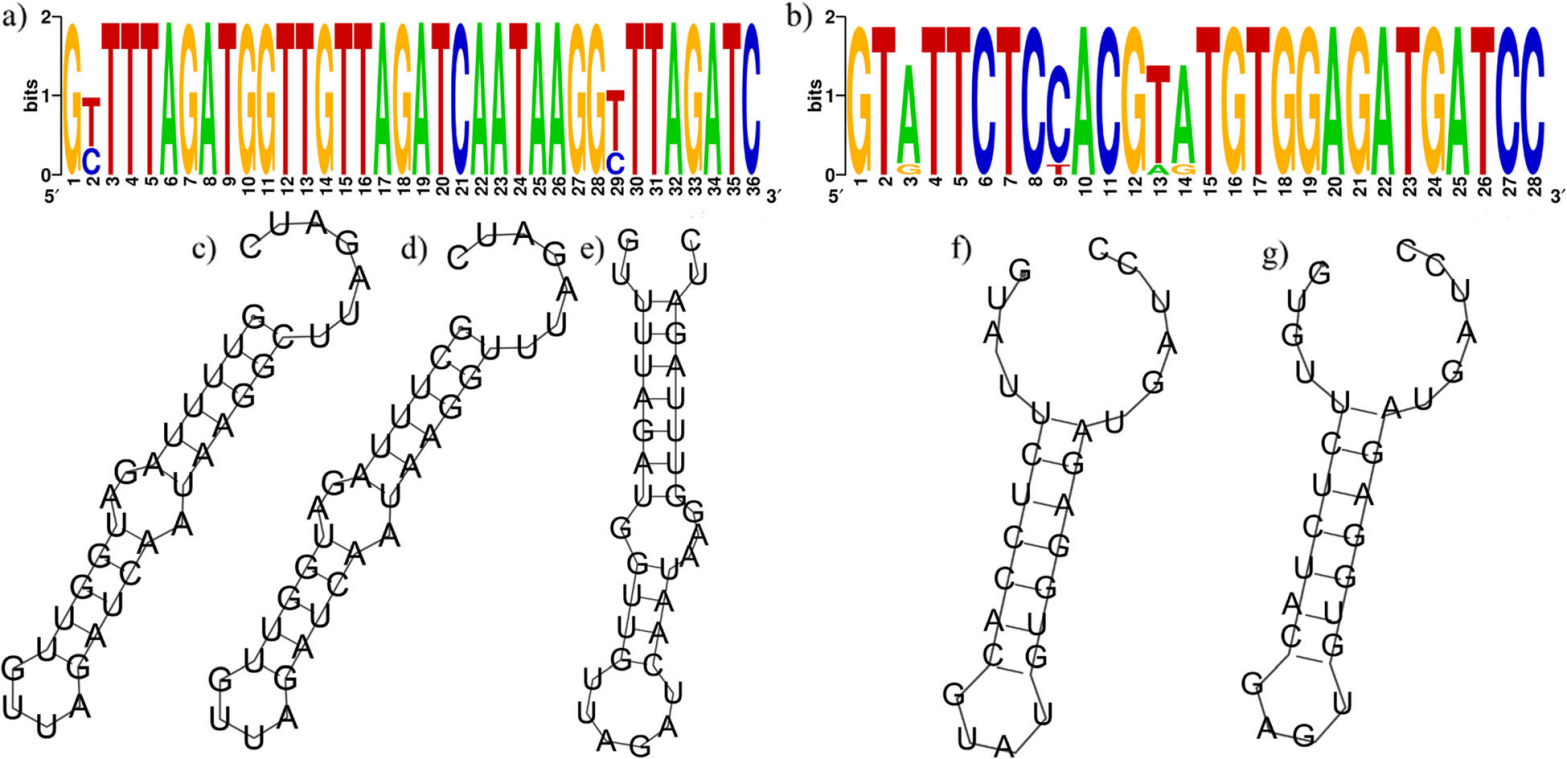
Features of DR sequences of CRISPR loci in *L. paragasseri* and *L. gasseri*. **a** The sequence of consensus DR sequences within *L. paragasseri*. **b** The sequence of consensus DR sequences in *L. gasseri* strains. The height of the letters indicates the frequency of the corresponding base at that position. **c**–**e** Predicted RNA secondary structures of CRISPR DR in *L. paragasseri*. **f**–**g** Predicted RNA secondary structures of the CRISPR DR in *L. gasseri*

The features of all 60 CRISPR loci identified in *L. paragasseri* and *L. gaseseri* genomes are summarized in Table S3. The length of DRs were 36 nucleotides (nt) in 36 strains of *L. paragasseri* except FJSCZD2-L1, FHNFQ53-L2 and FHNXY18-L3, which had DR sequences with 26 nt. The 5′-terminal portion of DRs in *L. paragasseri* were composed of G (T/C) TTT and the DRs were weakly palindromic. The putative RNA secondary structure of the DRs in *L. paragasseri* contained two small loops (Fig. 7). The DRs of *L. paragasseri* shared two variable nucleotides at the 2nd and 29th site (C/T), and the difference affected the RNA secondary structures (Fig. 7). While two CRISPR loci in *L. gasseri* had different DR sequences and varied in length and content, in which most of them were 28 nt whereas *L. gasseri* FHNFQ56-L1 and FHNFQ57-L4 had a same DR as *L. pargasseri* (Additional file 3: Table S3). Further, spacer contents were uncovered for *L. paragasseri* and *L. gaseseri*, ranging from 3 to 22 CRISPR spacers (Additional file 3: Table S3). The number of spacers in *L. paragasseri* and *L. gasseri* were variable and it provided information about the immunity record.

### Distribution of Bacteriocin operons

Identifying bacteriocins in vitro can be a challenging task, however, in silico analysis of genomes for presence of bacteriocin operons could make screening bacteriocin efficient. BAGEL was used to identify the potential bacteriocin operons in the current study. Three hundred twenty-three putative class II bacteriocin and 91 putative class Bacteriolysin (formerly Class III Bacteriocins) operons were identified in all 92 genomes (Additional file 4: Table S4). Class II bacteriocins are small heat stable peptides further subdivided IIa, IIb, IIc and IId based on the structure and activity of the peptides [25]. *L. paragassseri* genomes contained various bacteriocins including Class IIa (pediocin), Class IIb (gassericin K7B and gassericin T), Class IIc (acidocin B and gassericin A), Class IId (bacteriocin-LS2chaina and bacteriocin-LS2chainb), and Bacteriolysin, whereas all the strains of *L. gasseri* only encoded bacteriocin-helveticin-J (Bacteriolysin) except *L. gasseri* FHNFQ57-L4, which contained both bacteriocin-helveticin-J and pediocin operons.

Interestingly, gassericin K7B and gassericin T operons co-occured in 43 strains of *L. paragasseri*, and bacteriocin-LS2chaina and bacteriocin-LS2chainb co-occured in 67 strains of *L. paragasseri*. Sixteen gassericin A, 31 acidocin B, 69 pediocin and 78 bacteriocin-helveticin-J operons were also predicted in *L. paragasseri* indicating that helveticin homolog operons were more frequent than other operons. In addition, only one enterolysin A operon was found in *L. paragasseri* FHNFQ29-L2, FGSYC41-L1 and *L. paragasseri* FJSWX6-L7 contained a helveticin J operon.

Furthermore, according to the results, among all the 79 strains of *L. paragasseri*, at least one bacteriocin operon was found, in which 14 strains consisted of 8 bacteriocin operons including all types of Class II bacteriocin and bacteriocin-helveticin-J, and 17 strains contained 4 bacteriocin operons (pediocin, bacteriocin-LS2chaina, bacteriocin-LS2chainb and bacteriocin-helveticin-J), while *L. paragasseri* FHNFQ62-L6 was only predicted with bacteriocin-helveticin-J operon.

### The glycobiome of L. paragasseri and L. gasseri

**Fig. 8:**
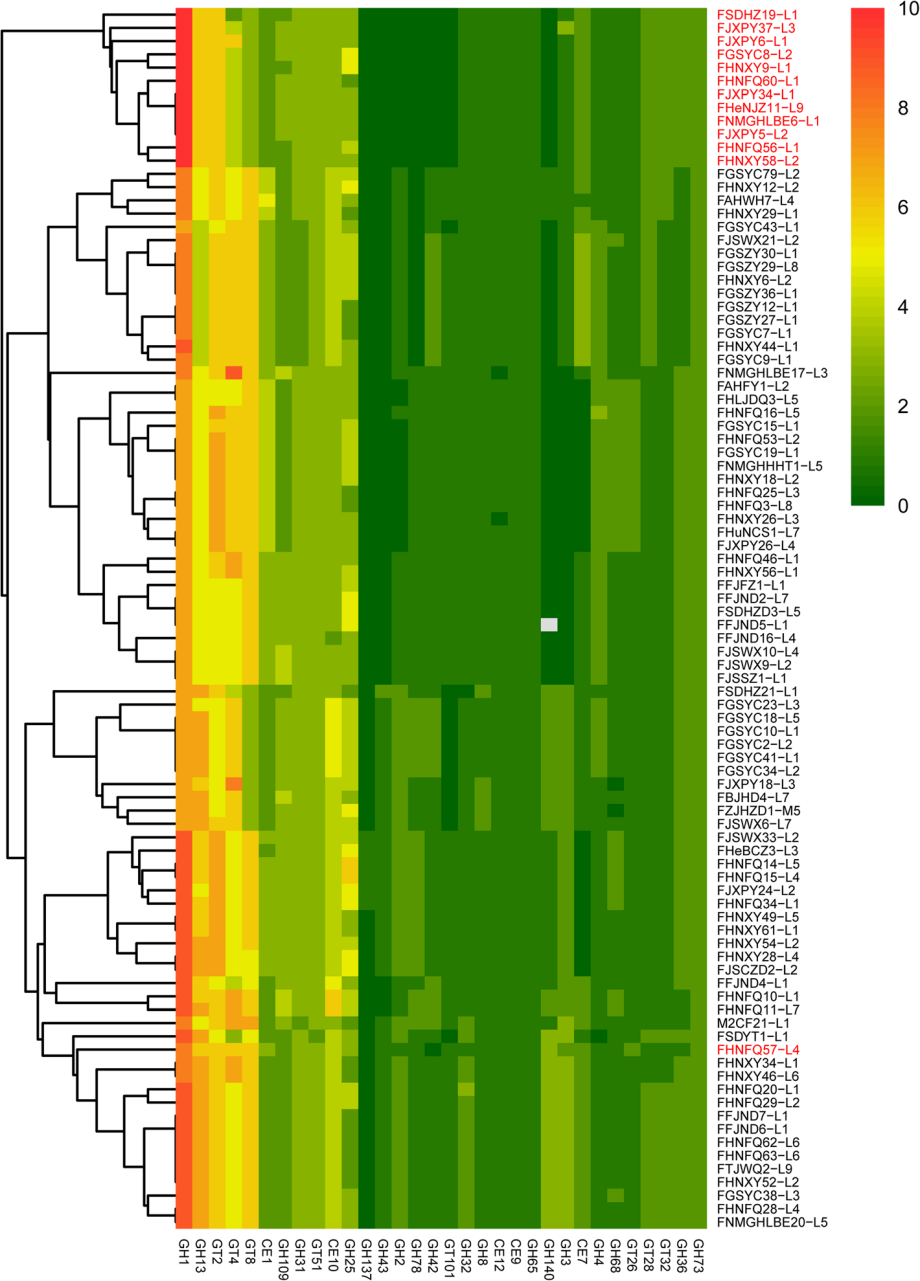
The distribution and number of GH, CE and GT family genes. Gene copy number was indicated by color ranging from green (absent) to red. The strain number in red and in black indicated *L. gasseri* and *L. paragasseri*, respectively

The earliest classifications of lactobacilli were based on their carbohydrate utilization patterns. In the current study, carbohydrate-active enzymes were analyzed by HMMER-3.1 and identified through carbohydrate-active enzyme (Cazy) database. Nineteen glycosyl hydrolase (GH) families, 7 glycosyl transferase (GT) families and 5 carbohydrate esterase (CE) families were predicted for each genome, and the distribution and abundance of GH, GT, CE family genes across the *L. paragasseri* and *L. gasseri* were showed by heatmap (Fig. 8).

The number of GH, GT and CE families’ enzymes were highly consistent in 12 strains of *L. gasseri* while variation was found in *L. paragasseri*. Among *L. paragasseri*, GH137 (β-L-arabinofuranosidase) was only predicted in 5 strains, GH65, GH73, GH8, CE9 and GT51 families showed exactly same and CE12 was detected in most of the strains except *L. paragasseri* FHNXY26-L3 and *L. paragasseri* FNMGHLBE17-L3. Notably, 12 strains of *L. paragasseri* including FNMGHHHT1-L5, FAHFY1-L2, FHNFQ25-L3, FHNXY18-L2, FHNXY26-L3, FHuNCS1-L1, FJXPY26-L4, FGSYC15-L1, FGSYC19-L1, FHLJDQ3-L5, FHNFQ3-L8 and FHNFQ53-L2, in which GH2 were absent, clustered a small branch in orthologous phylogenetic tree (Fig. 2). Similarly, the strains of FJSWX21-L2, FAHFY7-L4, FGSYC7-L1, FGSYC43-L1, FGSYC79-L2, FGSZY12-L1, FGSZY27-L1, FGSZY29-L8, FHNXY6-L2, FHNXY12-L2, FHNXY29-L1, FGSZY30-L1, FHNXY44-L1 and FGSZY36-L1, in which GH78 were absent, also formed a single clade. The number of GH, GT and CE families’ enzymes from Zhangye (Gansu Province) were completely consistent.

Twelve strains of *L. gasseri* formed a single clade using hierarchical clustering method (Fig. 8). Both species of *L. gasseri* and *L. paragasseri* appeared to contain a consistent GH65, GH73 and GT51 (murein polymerase) families, while GH42 family (β-galactosidase and α-L-arabinopyranosidase) was only found in *L. paragasseri*. Additionally, the gene number of GT8 (α-transferase) family in *L. gasseri* was less than that in *L. paragasseri*. The results revealed that carbohydrate utilization patterns of *L. gasseri* differed from *L. paragasseri*. Carbohydrate-active enzymes abundance in *L. paragasseri* showed high diversity, but the difference was not a result of gender and age difference, and may be associated with diet habits of the host individual. Diversity do not correlate with gender and age and could be result of sugar diet habits of the host individual.

## Discussion

NGS technologies have made sequencing easier to get high-quality bacterial genomes, and provides the possibility to better understand the genomic diversity within some genus [29]. In this study, genome sequences for 92 strains from human feces, which were preliminary identified as *L. gasseri* by 16S rDNA sequencing, combined with two publicly available genomes *L. gasseri* ATCC33323 and *L. paragasser*i K7, were further analysed. ANI values of 94 draft genomes were calculated through pairwise comparison at the 95% threshold, together with phylogenetic analysis based on orthologous genes and house-keeping genes (*pheS* and *groEL*) were performed to ensure the species affiliations and eliminated the mislabeled genomes only using ANI [30]. Seventy-nine strains were determined as *L. paragasseri*, and the remained 13 (14%) strains were *L. gasseri*, revealing that the most (86%) of isolates initially identified as *L. gasser*i by 16S rDNA sequencing were *L. paragasseri.* The current results were highly in line with previous publication by Tanizawa and colleagues [16], in which they reported that a large portion of genomes currently labelled as *L. gasseri* in the public database should be re-classified as *L. paragasseri* based on whole-genome sequence analyses as well. All those results indicated that *L. gasseri* and *L. paragesseri* are sister taxon with high similarity but not the same species, and the cultivable “*L. gasser*i” isolated from environment actually contained both *L. gasseri* and *L. paragasseri* species, which might be the reason for the high intraspecies diversity among “*L. gasseri*” exhibited. Meanwhile, *groEL*, a robust single-gene phylogenetic marker for *Lactobacillus* species identification [31], could serve as a marker to distinguish *L. paragasseri* and *L. gasseri*. Our current results provide a basis for distinguishing the two species by genotype. *L. gasseri* and *L. paragasseri* had no preference to colonize the female or male subjects, and the strains distribution had no trend on age neither infants nor adults. Nevertheless, a high intraspecies diversity in *L. paragasseri* may be caused by diet habits, health condition and others, which needs further research.

In general, the genome size of *L*. *paragasseri* and *L. gasseri* were smaller than other *Lactobacillus* species, which had an average size 1.96 Mb, while other *Lactobacillus* had a genome approximately 3.0 Mb, such as *L. paracasei* [20], *L. casei* [21], *Lactobacillus rhamnosus* [32]. Additionally, the G + C contents in *L. paragasseri* (34.9%) and *L. gasseri* (34.82%) were lower than that in other *Lactobacillus* species. For instance, average G + C contents were 38.96% in *L. reuteri* [19], 46.1–46.6% in *L. casei*, 46.5% in *L. paracasei* [20], and 46.5–46.8% in *L. rhamnosus* [33], and the average G + C content among lactobacilli genera is estimated at 42.4%. As previously found in bifidobacterial genomes, that the preferred start codon was ATG, also analysis of start codons in *L. paragasseri* and *L. gasseri* showed that they preferably used ATG as start codon [34].

Pan-genomes of *L. paragasseri* and *L. gasseri* were analysed, and the pan-genome size of the 80 strains among *L. paragasseri* and 14 strains of *L. gasseri* plus currently genome public strains of *L. gasseri* ATCC33323 and *L. paragasseri* K7 were 6535 and 2834 genes, respectively, and the core genomes were 1256 and 1375 genes, respectively, suggesting that open pan-genome within the *L. paragasseri* species and its pan-genome will increase if more *L. paragasseri* genomes were considered for the number of novel gene families and an open pan-genome implies that gene-exchanging within a species is higher [28]. But it could not conclude whether the pan-genome of *L. gasseri* was open or not because of limited number of sequenced genomes.

It has been reported that lactic acid bacteria are enriched resource for Type II CRISPR systems [35] and some previous studies on *L. gasseri* CRISPR-Cas reported that *L. gassseri* harboured type II-A CRISPR-Cas system with diversity in spacer content, and confirmed functionality [36]. However, the former results on “*L. gasseri*” might not be the real *L. gasseri*, as *L. paragasseri* was distinguished from *L. gasseri* recently, which might be mixed in the previous research. In the current result, *L. gasseri* and *L. paragasseri* were distinguished and separately, then were loaded for CRISAP-Cas analysis, respectively. The results showed that 39 of 79 *L. paragasseri* strains carried Type II systems and all the strains of *L. gasseri* harboured Type II and Type I CRISPR-Cas system (except FHNFQ57-L4), implying that both *L. paragasseri* and *L. gasseri* are main candidates for gene editing and cleavage of lytic bacteriophages in food industry. In the current study we found that *Cas1*, *Cas2* and *Cas9* were widespread across both *L. paragasseri* and *L. gasseri* species, and the *L. gasseri* species had a second *Cas1* and *Cas2*, while the second *Cas1* and *Cas2* were clustered in a single clade through phylogenetic analyses. Similarity, the *Cas9* gene was different between the two species, suggesting that CRISPR-Cas could provide a unique basis for resolution at the species-level [37], and the CRISPR-Cas systems may contribute to the evolutionary segregation [33].

It has been reported that *L. gasseri* produces variety of bacteriocin to inhibit some pathogens. Screening bacteriocin in vitro was complex and difficult while in silico analysis could make it rapid, generally using BAGEL to identify the potential bacteriocin operons. In the current study, most of the *L. gasseri* strains only had a single bacteriocin operon (Bacteriocin_helveticin_J), while *L. paragasseri* showed a variety of bacteriocin operons belonging to class II such as gassericin K7B, gassericin T and gassericin A. With the current results, although bacteriocin was not separated and verified in vitro, we presume that the strains with the high-yielding of bacteriocin, which was commonly known as *L. gasseri*, should actually be *L. paragaseri* rather than *L. gasseri*. For instance, previously *L. gasseri* LA39 was reported to produce gassericin A [38] and *L. gasseri* SBT2055 [39] could produce gassericin T, according to our results, they might belong to *L. paragasseri* species instead of *L. gasseri*. To confirm our hypothesis, more *L. gasseri* strains should be isolated and screened for bacteriocin to verify.

In order to investigate *L. paragasseri* and *L. gasseri* carbohydrate-utilization capabilities, carbohydrate-active enzymes were predicted for all the strains and these families have predicted substrates and functional properties for each strain. Analyzing the abundance of Cazy revealed that carbohydrate utilization patterns of *L. gasseri* significantly distinguished with *L. paragasseri* in genotype, which provided foundation for fermentation experiment with unique carbon sources. Moreover, 10.83% core genes had predicted function of carbohydrate transport and metabolism, which is the reason for strains diversity and separation.

## Conclusion

Ninety two strains isolated from Chinese subjects were initially identified as *L. gasseri* by 16S rDNA sequencing, while based on whole-genome analyses they were reclassified. According to ANI values and phylogenetic analysis based on both orthologous genes and house-keeping genes, 13 strains and 79 strains were reclassified as *L. gasseri* and *L. paragasseri*, respectively, which revealed a new species-level taxa from Chinese subjects. Pan-genome structure for *L. paragasseri* was open, meanwhile, *L. paragasseri* had a supragenome about 3.3 times larger than the average genome size of individual strains. After species reclassification, genetic features CRISPR-Cas systems, bacteriocin, and carbohydrate-active enzymes were analysed, revealing differences in the genomic characteristics of *L. paragasseri* and *L. gasseri* strains isolated from human feces and mine potential probiotic characteristics in the two species. To our knowledge, this is the first study to investigate pan/core-genome of *L. gasseri* and *L. paragasseri*, compared the genetic features between the two species.

## Methods

### Isolation of strains, genome sequencing and data assembly

Ninety-two strains isolated from adult and infant feces from different regions in China were listed in Table 1. Strains were selected in *Lactobacillus* selective medium (LBS) [4] and incubated at 37 °C in an anaerobic atmosphere (10% H_2_, 10% CO_2_, and 80% N_2_) in a anaerobic workstation (AW400TG, Electrotek Scientific Ltd., West Yorkshire, UK) for 18-24 h and 16S rRNA genes were sequenced for species identification. All the identified *L. gasseri* strains were stocked at -80 °C in 25% glycerol [40]. Draft genomes of all the 92 *L. gasseri* strains were sequenced via Illumina Hiseq× 10 platform (Majorbio BioTech Co, Shanghai, China), which generated 2 × 150 bp paired-end libraries and construct a paired-end library with an average read length of about 400 bp. It used double-end sequencing, which single-ended sequencing reads were 150 bp. The reads were assembled by SOAPde-novo and local inner gaps were filled by using the software GapCloser [41]. Two publicly available genomes (*L. gasseri* ATCC33323 [26] and *L. gasseri* K7 [27]) from National Centre for Biotechnology Information (https://www.ncbi.nlm.nih.gov/) were used for comparison and the latter one has recently been re-classified as *L. paragasseri* [16].

### Average nucleotide identity (ANI) values

ANI between any two genomes was calculated using python script (https://github.com/widdowquinn/pyani) [42] and the resulting matrix was clustered and visualized using R packages heatmap software [43].

### Phylogenetic analyses

All the genomic DNA were translated to protein sequences by EMBOSS-6.6.0 [44]. OrthoMCL1.4 was used to cluster orthologous genes and extracted all orthologous proteins sequences of 94 strains. All orthologous proteins were aligned using MAFFT-7.313 software [45] and phylogenetic trees were constructed using the python script (https://github.com/jvollme/fasta2phylip) and the supertree was modified using Evolgenius (http://www.evolgenius.info/evolview/). The house-keeping genes, *pheS* [46] and *groEL* [47], were extracted from the genomes using BLAST (Version 2.2.31+) [48], and the multiple alignments were carried out through Cluster-W (default parameters), and the single gene neighbor-joining trees were built by MEGA 6.0 [49], with bootstrap by a self-test of 1000 resampling.

### General feature predictions and annotation

The G + C content and start codon of each genome were predicted with Glimmer 3.02 [50] (http://ccb.jhu.edu/software/glimmer) prediction software. Transfer RNA (tRNA) was identified using tRNAscan-SE 2.0 [51] (http://lowelab.ucsc.edu/tRNAscan-SE/). Open Reading Frame (ORF) prediction was performed with Glimmer3.02 and ORFs were annotated by BLASTP analysis against the non-redundant protein databases created by BLASTP based on NCBI. Functions of the genome-encoded proteins were categorized based on clusters of orthologous groups (COG) (http://www.ncbi.nlm.nih.gov/COG/) assignments.

### Pan/core-genome analysis

Pan-genome computation for *L. paragasseri* and *L. gasseri* genomes was performed using the PGAP-1.2.1, which analysed multiple genomes based on protein sequences, nucleotide sequences and annotation information, and performed the analysis according to the Heap’s law pan-genome model [17, 52]. The ORF content of each genome was organised in functional gene clusters via the Gene Family method and a pan-genome profile was then built.

### CRISPR identification and characterization of isolated strains

The CRISPR (clustered regularly interspaced short palindromic repeats) regions and CRISPR-associated (Cas) proteins were identified by CRISPRCasFinder [53] (https://crisprcas.i2bc.paris-saclay.fr/CrisprCasFinder), and the CRISPR subtypes designation was based on the signature of Cas proteins [54]. MEGA6.0 was used to perform multiple sequence alignments, and neighbor-joining trees based on Cas1, Cas2 and Cas9 were bulit. The sequence of conserved direct repeats (DRs) were visualized by WebLogo (http://weblogo.berkeley.edu/). RNA secondary structure of DRs was performed by RNAfold web server with default arguments (http://rna.tbi.univie.ac.at/cgi-bin/RNAWebSuite/).

### Bacteriocin identification

The bacteriocin mining tool BAGEL3 was used to mine genomes for putative bacteriocin operons [55]. To determine the bacteriocins pre-identified by BAGEL3, BLASTP was secondly used to search each putative bacteriocin peptide against those pre-identified bacteriocins from BAGEL screening, and only the consistent results from both analysis were recognized as truly identified bacteriocin.

### The *L. gasseri* glycobiome

Analysis of the families of carbohydrate-active enzymes was carried out by using HMMER-3.1 (http://hmmer.org/) and with below a threshold cutoff of 1e-05. The copy number of the verified enzymes were summarized in a heatmap with hierarchical clustering method and Pearson distance [35].

## Supplementary information

**Additional file1: Table S1.** Cluster of Orthologous Groups (COGs) classification of *L. paragasseri*

**Additional file2: Table S2.** Cluster of Orthologous Groups (COGs) classification of *L. gasseri*

**Additional file3: Table S3.** CRISPR-Cas systems in *L. paragasseri* and *L. gasseri* strains

**Additional file4: Table S4.** Bacteriocin Operons in *L. paragasseri* and *L. gasseri*

**Additional file5: Figure S1.** Functional assignment of the *L. paragassei* (a) and *L. gasseri* core genome based on the COG database

## Data Availability

The genome datasets used during the current study are available from the corresponding author on reasonable request.

## Abbreviations

ANI: Average nucleotide identity
BLAST: Basic alignment search tool
Cazy: Carbohydrate-active enzyme
CE: Carbohydrate esterase
COG: Clusters of orthologous groups
GH: Glycosyl hydrolase
GT: Glycosyl transferase
NGS: Next generation sequencing
nt: Nucleotides
ORF: Open Reading Frames
Rep-PCR: Repetitive element-PCR
