# Supplementary material for: Comparative analysis of Lactobacillus gasseri from Chinese subjects reveals a new species-level taxa
Source: BMC Genomics. 2020 Feb 3;21:119. doi: 10.1186/s12864-020-6527-y (PMC6998098; doi:10.1186/s12864-020-6527-y)
Supplement: Supplementary file 1 — Additional file1: Table S1. Cluster of Orthologous Groups (COGs) classification of L. paragasseri [file 12864_2020_6527_MOESM1_ESM.doc]

**Table S1 Cluster of Orthologous Groups (COGs) classification of L.paragasseri**

| **strain** | **[C]** | **[D]** | **[E]** | **[F]** | **[G]** | **[H]** | **[I]** | **[J]** | **[K]** | **[L]** | **[M]** | **[N]** | **[O]** | **[P]** | **[Q]** | **[R]** | **[S]** | **[T]** | **[U]** | **[V]** |
| --- | --- | --- | --- | --- | --- | --- | --- | --- | --- | --- | --- | --- | --- | --- | --- | --- | --- | --- | --- | --- |
| FAHFY1-L2 | 53 | 23 | 99 | 60 | 157 | 30 | 34 | 137 | 117 | 115 | 88 | 5 | 41 | 59 | 6 | 159 | 149 | 29 | 15 | 29 |
| FAHFY7-L4 | 53 | 21 | 98 | 58 | 173 | 31 | 29 | 137 | 116 | 104 | 92 | 5 | 42 | 61 | 6 | 164 | 145 | 31 | 16 | 31 |
| FBJHD4-L7 | 53 | 22 | 97 | 55 | 165 | 29 | 35 | 137 | 119 | 113 | 86 | 5 | 39 | 63 | 7 | 167 | 147 | 30 | 13 | 30 |
| FFJND2-L7 | 54 | 20 | 100 | 58 | 161 | 27 | 29 | 137 | 128 | 115 | 96 | 5 | 39 | 60 | 6 | 161 | 154 | 32 | 18 | 32 |
| FGSYC10-L1 | 55 | 22 | 94 | 55 | 173 | 29 | 34 | 136 | 125 | 125 | 89 | 5 | 40 | 63 | 7 | 168 | 152 | 33 | 15 | 62 |
| FGSYC15-L1 | 53 | 23 | 99 | 60 | 157 | 29 | 33 | 137 | 122 | 120 | 93 | 5 | 40 | 59 | 6 | 162 | 150 | 31 | 17 | 55 |
| FGSYC18-L5 | 55 | 21 | 95 | 55 | 173 | 29 | 34 | 137 | 126 | 117 | 87 | 5 | 40 | 62 | 7 | 168 | 151 | 32 | 13 | 62 |
| FGSYC19-L1 | 53 | 23 | 99 | 56 | 154 | 30 | 33 | 136 | 117 | 117 | 98 | 5 | 41 | 60 | 6 | 159 | 148 | 32 | 15 | 55 |
| FGSYC2-L2 | 56 | 21 | 94 | 54 | 174 | 28 | 34 | 136 | 124 | 119 | 87 | 5 | 40 | 60 | 6 | 168 | 151 | 32 | 15 | 58 |
| FGSYC23-L3 | 55 | 21 | 94 | 55 | 173 | 29 | 34 | 136 | 124 | 117 | 86 | 5 | 40 | 62 | 7 | 168 | 152 | 32 | 13 | 61 |
| FGSYC34-L2 | 55 | 21 | 94 | 55 | 173 | 29 | 34 | 137 | 125 | 118 | 88 | 5 | 40 | 63 | 7 | 168 | 152 | 32 | 13 | 61 |
| FGSYC38-L3 | 53 | 21 | 96 | 60 | 184 | 31 | 33 | 136 | 126 | 110 | 95 | 5 | 40 | 63 | 7 | 160 | 145 | 31 | 15 | 48 |
| FGSYC41-L1 | 55 | 21 | 95 | 55 | 173 | 29 | 34 | 136 | 125 | 122 | 90 | 5 | 40 | 63 | 7 | 168 | 151 | 32 | 13 | 61 |
| FGSYC43-L1 | 53 | 21 | 101 | 59 | 162 | 31 | 33 | 136 | 128 | 113 | 95 | 5 | 44 | 63 | 7 | 161 | 152 | 31 | 15 | 47 |
| FGSYC7-L1 | 53 | 21 | 103 | 59 | 162 | 31 | 33 | 137 | 121 | 106 | 96 | 5 | 43 | 63 | 8 | 159 | 147 | 31 | 15 | 45 |
| FGSYC79-L2 | 54 | 22 | 101 | 58 | 173 | 31 | 29 | 136 | 118 | 118 | 96 | 5 | 43 | 61 | 7 | 163 | 152 | 32 | 19 | 50 |
| FGSYC9-L1 | 54 | 21 | 102 | 59 | 164 | 31 | 33 | 136 | 123 | 111 | 99 | 5 | 43 | 63 | 8 | 160 | 152 | 31 | 15 | 45 |
| FGSZY12-L1 | 53 | 21 | 103 | 59 | 162 | 31 | 33 | 136 | 124 | 110 | 94 | 5 | 44 | 63 | 8 | 165 | 147 | 31 | 15 | 44 |
| FGSZY27-L1 | 53 | 21 | 103 | 59 | 162 | 31 | 33 | 136 | 125 | 113 | 99 | 5 | 44 | 63 | 8 | 165 | 148 | 32 | 15 | 44 |
| FGSZY29-L8 | 52 | 21 | 102 | 59 | 163 | 30 | 33 | 136 | 126 | 117 | 100 | 5 | 44 | 60 | 7 | 163 | 154 | 31 | 17 | 49 |
| FGSZY30-L1 | 52 | 21 | 102 | 60 | 162 | 31 | 33 | 136 | 124 | 113 | 101 | 5 | 44 | 63 | 8 | 162 | 153 | 32 | 15 | 48 |
| FGSZY36-L1 | 52 | 21 | 102 | 59 | 162 | 30 | 33 | 136 | 124 | 117 | 100 | 5 | 44 | 60 | 7 | 162 | 155 | 32 | 15 | 49 |
| FHeBCZ3-L3 | 57 | 21 | 103 | 62 | 178 | 26 | 30 | 137 | 127 | 112 | 94 | 6 | 45 | 61 | 6 | 172 | 147 | 30 | 14 | 30 |
| FHLJDQ3-L5 | 54 | 23 | 99 | 59 | 158 | 30 | 34 | 138 | 116 | 117 | 93 | 5 | 41 | 58 | 6 | 156 | 147 | 29 | 15 | 29 |
| FHNFQ10-L1 | 56 | 22 | 104 | 60 | 188 | 29 | 35 | 137 | 130 | 113 | 93 | 5 | 43 | 60 | 8 | 169 | 151 | 28 | 16 | 58 |
| FHNFQ11-L7 | 56 | 22 | 104 | 60 | 188 | 29 | 35 | 137 | 129 | 114 | 96 | 5 | 43 | 60 | 8 | 170 | 151 | 31 | 16 | 58 |
| FHNFQ14-L5 | 58 | 23 | 103 | 63 | 180 | 28 | 29 | 139 | 133 | 122 | 100 | 5 | 45 | 64 | 7 | 175 | 154 | 32 | 14 | 60 |
| FHNFQ15-L4 | 60 | 23 | 103 | 63 | 180 | 28 | 29 | 139 | 134 | 129 | 99 | 5 | 45 | 64 | 7 | 174 | 154 | 32 | 16 | 61 |
| FHNFQ16-L5 | 55 | 24 | 99 | 61 | 156 | 30 | 35 | 140 | 119 | 121 | 99 | 5 | 42 | 62 | 7 | 168 | 153 | 32 | 16 | 61 |
| FHNFQ20-L1 | 53 | 22 | 97 | 61 | 185 | 31 | 33 | 137 | 134 | 116 | 97 | 5 | 41 | 62 | 7 | 161 | 151 | 32 | 17 | 49 |
| FHNFQ25-L3 | 53 | 22 | 100 | 60 | 156 | 29 | 34 | 137 | 114 | 112 | 93 | 5 | 40 | 59 | 6 | 162 | 145 | 29 | 15 | 54 |
| FHNFQ28-L4 | 53 | 21 | 97 | 60 | 185 | 31 | 33 | 136 | 127 | 116 | 95 | 5 | 40 | 62 | 7 | 162 | 147 | 31 | 17 | 49 |
| FHNFQ29-L2 | 53 | 22 | 96 | 60 | 185 | 31 | 33 | 136 | 127 | 117 | 99 | 5 | 41 | 62 | 7 | 161 | 148 | 31 | 15 | 48 |
| FHNFQ3-L8 | 54 | 23 | 100 | 61 | 157 | 29 | 35 | 137 | 118 | 117 | 95 | 5 | 40 | 59 | 6 | 162 | 147 | 32 | 15 | 55 |
| FHNFQ34-L1 | 59 | 23 | 104 | 63 | 180 | 30 | 29 | 140 | 136 | 117 | 96 | 5 | 45 | 64 | 8 | 174 | 154 | 31 | 14 | 62 |
| FHNFQ46-L1 | 54 | 20 | 102 | 57 | 160 | 26 | 29 | 138 | 117 | 110 | 98 | 5 | 41 | 58 | 6 | 161 | 147 | 29 | 15 | 54 |
| FHNFQ53-L2 | 53 | 25 | 100 | 56 | 153 | 30 | 33 | 136 | 115 | 123 | 98 | 5 | 42 | 59 | 6 | 162 | 149 | 32 | 15 | 58 |
| FHNFQ62-L6 | 54 | 22 | 97 | 60 | 187 | 33 | 33 | 136 | 122 | 109 | 94 | 5 | 40 | 65 | 7 | 162 | 143 | 31 | 18 | 49 |
| FHNFQ63-L6 | 54 | 22 | 96 | 60 | 187 | 31 | 33 | 136 | 122 | 114 | 94 | 5 | 40 | 64 | 7 | 162 | 144 | 31 | 18 | 49 |
| FHNXY12-L2 | 53 | 21 | 98 | 58 | 174 | 32 | 29 | 136 | 125 | 112 | 95 | 5 | 42 | 63 | 7 | 165 | 150 | 33 | 16 | 47 |
| FHNXY18-L2 | 52 | 22 | 98 | 56 | 157 | 31 | 33 | 137 | 119 | 112 | 95 | 5 | 40 | 59 | 7 | 159 | 148 | 30 | 15 | 54 |
| FHNXY26-L3 | 53 | 22 | 101 | 60 | 157 | 30 | 33 | 137 | 116 | 113 | 93 | 5 | 41 | 59 | 6 | 162 | 149 | 29 | 16 | 56 |
| FHNXY28-L4 | 58 | 21 | 102 | 60 | 180 | 28 | 29 | 139 | 130 | 118 | 95 | 5 | 46 | 64 | 7 | 170 | 153 | 30 | 14 | 58 |
| FHNXY29-L1 | 54 | 21 | 100 | 58 | 174 | 34 | 29 | 137 | 116 | 109 | 92 | 5 | 44 | 65 | 7 | 163 | 143 | 31 | 17 | 49 |
| FHNXY34-L1 | 53 | 21 | 96 | 60 | 180 | 31 | 33 | 136 | 121 | 107 | 92 | 5 | 40 | 62 | 7 | 158 | 143 | 31 | 15 | 49 |
| FHNXY44-L1 | 54 | 23 | 101 | 58 | 162 | 32 | 33 | 136 | 123 | 110 | 96 | 5 | 42 | 63 | 8 | 164 | 149 | 31 | 17 | 49 |
| FHNXY46-L6 | 54 | 21 | 96 | 60 | 180 | 32 | 33 | 136 | 124 | 108 | 93 | 5 | 40 | 62 | 7 | 157 | 146 | 31 | 15 | 48 |
| FHNXY49-L5 | 58 | 21 | 103 | 60 | 179 | 27 | 29 | 138 | 124 | 108 | 93 | 5 | 45 | 65 | 7 | 168 | 145 | 29 | 14 | 58 |
| FHNXY52-L2 | 54 | 21 | 96 | 59 | 184 | 30 | 33 | 137 | 125 | 108 | 93 | 5 | 40 | 60 | 6 | 159 | 142 | 31 | 15 | 48 |
| FHNXY54-L2 | 58 | 23 | 104 | 59 | 180 | 27 | 29 | 137 | 125 | 111 | 94 | 5 | 45 | 61 | 7 | 168 | 149 | 30 | 16 | 59 |
| FHNXY56-L1 | 54 | 22 | 101 | 61 | 161 | 27 | 29 | 137 | 120 | 119 | 96 | 5 | 41 | 60 | 7 | 162 | 152 | 30 | 15 | 59 |
| FHNXY6-L2 | 54 | 21 | 101 | 58 | 160 | 32 | 33 | 136 | 129 | 109 | 94 | 5 | 42 | 63 | 8 | 166 | 154 | 38 | 15 | 48 |
| FHNXY61-L1 | 59 | 22 | 102 | 60 | 181 | 28 | 30 | 139 | 128 | 113 | 92 | 5 | 45 | 64 | 7 | 168 | 149 | 30 | 14 | 58 |
| FHuNCS1-L1 | 54 | 23 | 99 | 61 | 160 | 31 | 33 | 139 | 119 | 120 | 94 | 5 | 41 | 62 | 7 | 162 | 151 | 29 | 15 | 29 |
| FFJFZ1-L2 | 54 | 21 | 102 | 57 | 162 | 27 | 31 | 137 | 116 | 118 | 90 | 5 | 40 | 58 | 7 | 159 | 154 | 29 | 18 | 29 |
| FFJND16-L4 | 55 | 20 | 101 | 58 | 160 | 27 | 28 | 137 | 116 | 110 | 89 | 5 | 40 | 60 | 6 | 157 | 150 | 29 | 16 | 29 |
| FFJND4-L5 | 54 | 21 | 98 | 59 | 189 | 30 | 33 | 136 | 125 | 106 | 83 | 5 | 40 | 59 | 6 | 159 | 145 | 30 | 16 | 30 |
| FFJND5-L1 | 54 | 20 | 100 | 58 | 161 | 27 | 29 | 137 | 128 | 115 | 91 | 5 | 39 | 60 | 6 | 161 | 154 | 32 | 18 | 32 |
| FFJND6-L1 | 53 | 21 | 96 | 59 | 184 | 30 | 33 | 136 | 123 | 109 | 94 | 5 | 41 | 60 | 6 | 160 | 146 | 31 | 15 | 31 |
| FFJND7-L1 | 53 | 21 | 96 | 59 | 184 | 30 | 33 | 136 | 123 | 109 | 94 | 5 | 41 | 60 | 6 | 160 | 145 | 31 | 15 | 31 |
| FJSWX6-L7 | 55 | 21 | 96 | 54 | 165 | 28 | 33 | 137 | 129 | 123 | 88 | 5 | 41 | 62 | 6 | 171 | 150 | 30 | 15 | 30 |
| FJXPY18-L3 | 53 | 21 | 95 | 55 | 166 | 29 | 33 | 135 | 122 | 114 | 95 | 5 | 40 | 62 | 7 | 167 | 149 | 29 | 13 | 29 |
| FJXPY24-L2 | 58 | 22 | 102 | 63 | 180 | 28 | 29 | 139 | 132 | 120 | 95 | 5 | 45 | 64 | 7 | 171 | 151 | 30 | 16 | 30 |
| FJXPY26-L4 | 55 | 24 | 100 | 61 | 158 | 32 | 33 | 138 | 120 | 124 | 96 | 5 | 41 | 62 | 7 | 162 | 152 | 29 | 15 | 29 |
| FNMGHHHT1-L5 | 53 | 22 | 99 | 53 | 158 | 30 | 33 | 138 | 119 | 116 | 93 | 5 | 41 | 59 | 6 | 160 | 148 | 30 | 15 | 30 |
| FNMGHLBE17-L3 | 54 | 22 | 100 | 57 | 163 | 28 | 31 | 137 | 119 | 118 | 96 | 5 | 42 | 60 | 7 | 163 | 151 | 29 | 16 | 29 |
| FNMGHLBE20-L5 | 54 | 23 | 95 | 61 | 183 | 31 | 34 | 136 | 125 | 113 | 94 | 5 | 41 | 64 | 7 | 162 | 147 | 31 | 15 | 31 |
| FSDHZ21-L1 | 54 | 21 | 95 | 54 | 167 | 29 | 33 | 137 | 124 | 117 | 89 | 5 | 40 | 59 | 6 | 170 | 151 | 30 | 15 | 30 |
| FTJWQ2-L9 | 56 | 21 | 96 | 61 | 186 | 33 | 45 | 136 | 127 | 115 | 92 | 5 | 40 | 61 | 7 | 160 | 146 | 32 | 15 | 32 |
| FJSCZD2-L1 | 56 | 20 | 102 | 58 | 162 | 26 | 30 | 137 | 123 | 117 | 88 | 5 | 41 | 58 | 5 | 164 | 157 | 32 | 18 | 32 |
| FJSSZ1-L1 | 54 | 20 | 101 | 57 | 161 | 27 | 33 | 137 | 118 | 108 | 89 | 5 | 41 | 60 | 6 | 161 | 149 | 29 | 16 | 29 |
| FJSWX10-L4 | 55 | 21 | 101 | 58 | 160 | 27 | 33 | 138 | 120 | 111 | 90 | 5 | 41 | 60 | 7 | 162 | 150 | 29 | 16 | 29 |
| FJSWX21-L2 | 53 | 21 | 101 | 58 | 161 | 31 | 33 | 137 | 122 | 109 | 97 | 5 | 42 | 63 | 8 | 163 | 154 | 34 | 15 | 34 |
| FJSWX33-L2 | 57 | 23 | 103 | 62 | 179 | 28 | 29 | 137 | 125 | 113 | 95 | 5 | 44 | 61 | 6 | 167 | 147 | 32 | 16 | 32 |
| FJSWX9-L2 | 55 | 21 | 101 | 58 | 161 | 28 | 33 | 137 | 119 | 111 | 91 | 5 | 41 | 60 | 7 | 162 | 150 | 29 | 16 | 29 |
| M2CF21-L1 | 58 | 22 | 100 | 58 | 177 | 29 | 29 | 138 | 126 | 105 | 95 | 6 | 45 | 60 | 9 | 174 | 153 | 27 | 15 | 27 |
| FSDHZD3-L5 | 57 | 20 | 101 | 57 | 159 | 27 | 29 | 138 | 125 | 117 | 93 | 5 | 41 | 57 | 6 | 158 | 158 | 30 | 18 | 30 |
| FSDYT1-L1 | 53 | 21 | 95 | 60 | 184 | 30 | 33 | 137 | 126 | 113 | 96 | 5 | 40 | 59 | 5 | 160 | 150 | 32 | 17 | 32 |
| FZJHZD1-M5 | 53 | 21 | 95 | 56 | 170 | 29 | 37 | 135 | 127 | 119 | 90 | 5 | 40 | 62 | 8 | 169 | 151 | 31 | 13 | 31 |

[C]:Energy production and conversion ;[D]:Cell cycle control, cell division, chromosome partitioning ;[E]:Amino acid transport and metabolism;

[F]:Nucleotide transport and metabolism ;[G]:Carbohydrate transport and metabolism ;[H]:Coenzyme transport and metabolism;

[I]:Lipid transport and metabolism ;[J]:Translation, ribosomal structure and biogenesis ;[K]:Transcription ;[L]:Replication, recombination and repair ;

[M]:Cell wall/membrane/envelope biogenesis ;[N]:Cell motility ;[O]:Posttranslational modification, protein turnover, chaperones;

[P]:Inorganic ion transport and metabolism ;[Q]:Secondary metabolites biosynthesis, transport and catabolism ;[R]:General function prediction only ;

[S]:Function unknown ;[T]:Signal transduction mechanisms ;[U]:Intracellular trafficking, secretion, and vesicular transport ;[V]:Defense mechanisms ;
