# Supplementary material for: Comparative analysis of Lactobacillus gasseri from Chinese subjects reveals a new species-level taxa
Source: BMC Genomics. 2020 Feb 3;21:119. doi: 10.1186/s12864-020-6527-y (PMC6998098; doi:10.1186/s12864-020-6527-y)
Supplement: Supplementary file 2 — Additional file2: Table S2. Cluster of Orthologous Groups (COGs) classification of L. gasseri [file 12864_2020_6527_MOESM2_ESM.doc]

**Table S2 Cluster of Orthologous Groups (COGs) classification of L.gasseri**

| **strain** | **[C]** | **[D]** | **[E]** | **[F]** | **[G]** | **[H]** | **[I]** | **[J]** | **[K]** | **[L]** | **[M]** | **[N]** | **[O]** | **[P]** | **[Q]** | **[R]** | **[S]** | **[T]** | **[U]** | **[V]** |
| --- | --- | --- | --- | --- | --- | --- | --- | --- | --- | --- | --- | --- | --- | --- | --- | --- | --- | --- | --- | --- |
| FGSYC8-L2 | 54 | 22 | 93 | 57 | 182 | 28 | 29 | 136 | 130 | 127 | 91 | 5 | 46 | 59 | 7 | 173 | 160 | 31 | 13 | 54 |
| FHeNJZ11-L9 | 54 | 22 | 93 | 57 | 184 | 29 | 29 | 136 | 123 | 117 | 91 | 5 | 44 | 59 | 7 | 164 | 152 | 30 | 13 | 30 |
| FHNFQ56-L1 | 54 | 21 | 93 | 57 | 185 | 29 | 28 | 136 | 121 | 120 | 91 | 5 | 47 | 60 | 7 | 169 | 155 | 30 | 13 | 51 |
| FHNFQ57-L4 | 51 | 20 | 85 | 54 | 174 | 27 | 31 | 136 | 116 | 102 | 92 | 5 | 41 | 59 | 4 | 153 | 151 | 31 | 15 | 52 |
| FHNFQ60-L1 | 53 | 22 | 92 | 58 | 186 | 29 | 29 | 135 | 121 | 114 | 88 | 5 | 44 | 60 | 7 | 165 | 149 | 30 | 13 | 49 |
| FHNXY58-L2 | 54 | 21 | 92 | 57 | 185 | 30 | 27 | 136 | 120 | 113 | 88 | 5 | 44 | 59 | 7 | 161 | 147 | 30 | 13 | 48 |
| FHNXY9-L1 | 55 | 22 | 92 | 57 | 186 | 29 | 28 | 136 | 126 | 122 | 89 | 5 | 45 | 59 | 7 | 170 | 154 | 31 | 15 | 48 |
| FJXPY34-L1 | 54 | 22 | 94 | 57 | 183 | 28 | 29 | 136 | 123 | 114 | 89 | 5 | 44 | 60 | 7 | 167 | 151 | 31 | 13 | 31 |
| FJXPY37-L3 | 55 | 23 | 94 | 57 | 182 | 28 | 30 | 136 | 121 | 119 | 91 | 5 | 44 | 59 | 7 | 166 | 151 | 31 | 15 | 31 |
| FJXPY5-L2 | 54 | 22 | 93 | 57 | 185 | 28 | 33 | 136 | 128 | 127 | 92 | 5 | 45 | 60 | 8 | 168 | 151 | 31 | 15 | 31 |
| FJXPY6-L1 | 55 | 22 | 93 | 57 | 184 | 28 | 30 | 136 | 126 | 124 | 95 | 5 | 44 | 59 | 7 | 167 | 154 | 32 | 15 | 32 |
| FNMGHLBE6-L1 | 55 | 23 | 96 | 58 | 187 | 28 | 30 | 136 | 122 | 116 | 89 | 5 | 43 | 61 | 8 | 169 | 151 | 30 | 13 | 30 |

[C]:Energy production and conversion ;[D]:Cell cycle control, cell division, chromosome partitioning ;[E]:Amino acid transport and metabolism;

[F]:Nucleotide transport and metabolism ;[G]:Carbohydrate transport and metabolism ;[H]:Coenzyme transport and metabolism;

[I]:Lipid transport and metabolism ;[J]:Translation, ribosomal structure and biogenesis ;[K]:Transcription ;[L]:Replication, recombination and repair ;

[M]:Cell wall/membrane/envelope biogenesis ;[N]:Cell motility ;[O]:Posttranslational modification, protein turnover, chaperones;

[P]:Inorganic ion transport and metabolism ;[Q]:Secondary metabolites biosynthesis, transport and catabolism ;[R]:General function prediction only ;

[S]:Function unknown ;[T]:Signal transduction mechanisms ;[U]:Intracellular trafficking, secretion, and vesicular transport ;[V]:Defense mechanisms ;
