# Supplementary material for: Comparative analysis of Lactobacillus gasseri from Chinese subjects reveals a new species-level taxa
Source: BMC Genomics. 2020 Feb 3;21:119. doi: 10.1186/s12864-020-6527-y (PMC6998098; doi:10.1186/s12864-020-6527-y)
Supplement: Supplementary file 4 — Additional file4: Table S4. Bacteriocin Operons in L. paragasseri and L. gasseri [file 12864_2020_6527_MOESM4_ESM.doc]

| **Table S4 Bacteriocin Operons in** L.paragasseri and L.gasseri | | | | | |
| --- | --- | --- | --- | --- | --- |
| **strains** | **Bacteriocin** | **Subclass** | **strains** | **Bacteriocin** | **Subclass** |
| FAHFY1-L2 | Pediocin | ClassIIa | FHNXY29-L1 | Bacteriocin_helveticin_J | Bacteriolysin |
|  | Acidocin_LF221B(GassericinK7B) | ClassIIb |  | Pediocin | ClassIIa |
|  | Gassericin_T | ClassIIb |  | Acidocin_LF221B(GassericinK7B) | ClassIIb |
|  | Acidocin_B | ClassIIc |  | Gassericin_T | ClassIIb |
|  | Gassericin_A | ClassIIc |  | bacteriocin_LS2chaina | ClassIId |
|  | bacteriocin_LS2chaina | ClassIId |  | bacteriocin_LS2chainb | ClassIId |
|  | bacteriocin_LS2chainb | ClassIId | FHNFQ46-L1 | Bacteriocin_helveticin_J | Bacteriolysin |
|  | Bacteriocin_helveticin_J | Bacteriolysin |  | Gassericin_T | ClassIIb |
| FAHFY7-L4 | bacteriocin_LS2chainb | ClassIId |  | Acidocin_LF221B(GassericinK7B) | ClassIIb |
|  | bacteriocin_LS2chaina | ClassIId |  | Pediocin | ClassIIa |
|  | Pediocin | ClassIIa |  | bacteriocin_LS2chainb | ClassIId |
|  | Acidocin_LF221B(GassericinK7B) | ClassIIb |  | bacteriocin_LS2chaina | ClassIId |
|  | Gassericin_T | ClassIIb |  | Gassericin_A | ClassIIc |
|  | Bacteriocin_helveticin_J | Bacteriolysin |  | Acidocin_B | ClassIIc |
| FBJHD4-L7 | Acidocin_B | ClassIIc | FHNFQ53-L2 | Acidocin_B | ClassIIc |
|  | Gassericin_A | ClassIIc |  | Gassericin_A | ClassIIc |
|  | Pediocin | ClassIIa |  | bacteriocin_LS2chaina | ClassIId |
|  | Bacteriocin_helveticin_J | Bacteriolysin |  | bacteriocin_LS2chainb | ClassIId |
| FFJND2-L7 | Bacteriocin_helveticin_J | Bacteriolysin |  | Bacteriocin_helveticin_J | Bacteriolysin |
|  | Gassericin_T | ClassIIb | FHNFQ62-L6 | Bacteriocin_helveticin_J | Bacteriolysin |
|  | Acidocin_LF221B(GassericinK7B) | ClassIIb | FHNFQ63-L6 | Pediocin | ClassIIa |
|  | Pediocin | ClassIIa |  | Bacteriocin_helveticin_J | Bacteriolysin |
|  | bacteriocin_LS2chaina | ClassIId |  | bacteriocin_LS2chaina | ClassIId |
|  | bacteriocin_LS2chainb | ClassIId |  | bacteriocin_LS2chainb | ClassIId |
|  | Acidocin_B | ClassIIc | FHNXY12-L2 | Pediocin | ClassIIa |
|  | Acidocin_B | ClassIIc |  | Acidocin_LF221B(GassericinK7B) | ClassIIb |
| FFJND16-L4 | bacteriocin_LS2chainb | ClassIId |  | Gassericin_T | ClassIIb |
|  | bacteriocin_LS2chaina | ClassIId |  | Bacteriocin_helveticin_J | Bacteriolysin |
|  | Acidocin_B | ClassIIc |  | bacteriocin_LS2chaina | ClassIId |
|  | Gassericin_A | ClassIIc |  | bacteriocin_LS2chainb | ClassIId |
|  | Bacteriocin_helveticin_J | Bacteriolysin | FHNXY18-L2 | Pediocin | ClassIIa |
|  | Pediocin | ClassIIa |  | Acidocin_B | ClassIIc |
| FFJFZ1-L2 | Acidocin_B | ClassIIc |  | Gassericin_A | ClassIIc |
|  | Gassericin_A | ClassIIc |  | bacteriocin_LS2chainb | ClassIId |
|  | Bacteriocin_helveticin_J | Bacteriolysin |  | bacteriocin_LS2chaina | ClassIId |
|  | Gassericin_T | ClassIIb |  | Bacteriocin_helveticin_J | Bacteriolysin |
|  | Acidocin_LF221B(GassericinK7B) | ClassIIb |  | Acidocin_LF221B(GassericinK7B) | ClassIIb |
|  | Pediocin | ClassIIa |  | Gassericin_T | ClassIIb |
|  | bacteriocin_LS2chaina | ClassIId | FHNXY26-L3 | Pediocin | ClassIIa |
|  | bacteriocin_LS2chainb | ClassIId |  | Acidocin_LF221B(GassericinK7B) | ClassIIb |
| FFJND4-L5 | Bacteriocin_helveticin_J | Bacteriolysin |  | Gassericin_T | ClassIIb |
|  | Gassericin_T | ClassIIb |  | Acidocin_B | ClassIIc |
|  | Acidocin_LF221B(GassericinK7B) | ClassIIb |  | bacteriocin_LS2chaina | ClassIId |
|  | Pediocin | ClassIIa |  | bacteriocin_LS2chainb | ClassIId |
| FFJND5-L1 | bacteriocin_LS2chainb | ClassIId |  | Bacteriocin_helveticin_J | Bacteriolysin |
|  | bacteriocin_LS2chaina | ClassIId | FHNXY28-L4 | Bacteriocin_helveticin_J | Bacteriolysin |
|  | Bacteriocin_helveticin_J | Bacteriolysin |  | bacteriocin_LS2chainb | ClassIId |
|  | Gassericin_T | ClassIIb |  | bacteriocin_LS2chaina | ClassIId |
|  | Acidocin_LF221B(GassericinK7B) | ClassIIb | FHNXY29-L1 | Pediocin | ClassIIa |
|  | Pediocin | ClassIIa |  | Acidocin_LF221B(GassericinK7B) | ClassIIb |
| FFJND6-L1 | Pediocin | ClassIIa |  | Gassericin_T | ClassIIb |
|  | Bacteriocin_helveticin_J | Bacteriolysin |  | Bacteriocin_helveticin_J | Bacteriolysin |
|  | bacteriocin_LS2chaina | ClassIId |  | bacteriocin_LS2chaina | ClassIId |
|  | bacteriocin_LS2chainb | ClassIId |  | bacteriocin_LS2chainb | ClassIId |
| FFJND7-L1 | Bacteriocin_helveticin_J | Bacteriolysin | FHNXY34-L1 | bacteriocin_LS2chaina | ClassIId |
|  | Pediocin | ClassIIa |  | bacteriocin_LS2chainb | ClassIId |
|  | bacteriocin_LS2chaina | ClassIId |  | Pediocin | ClassIIa |
|  | bacteriocin_LS2chainb | ClassIId |  | Bacteriocin_helveticin_J | Bacteriolysin |
| FGSYC10-L1 | Bacteriocin_helveticin_J | Bacteriolysin | FHNXY44-L1 | Gassericin_T | ClassIIb |
|  | Pediocin | ClassIIa |  | Acidocin_LF221B(GassericinK7B) | ClassIIb |
|  | Acidocin_B | ClassIIc |  | Pediocin | ClassIIa |
| FGSYC15-L1 | Acidocin_B | ClassIIc |  | bacteriocin_LS2chainb | ClassIId |
|  | Gassericin_T | ClassIIb |  | bacteriocin_LS2chaina | ClassIId |
|  | Acidocin_LF221B(GassericinK7B) | ClassIIb |  | Bacteriocin_helveticin_J | Bacteriolysin |
|  | Pediocin | ClassIIa | FHNXY46-L6 | Pediocin | ClassIIa |
|  | bacteriocin_LS2chaina | ClassIId |  | Bacteriocin_helveticin_J | Bacteriolysin |
|  | bacteriocin_LS2chainb | ClassIId |  | bacteriocin_LS2chaina | ClassIId |
|  | Bacteriocin_helveticin_J | Bacteriolysin |  | bacteriocin_LS2chainb | ClassIId |
| FGSYC18-L5 | Bacteriocin_helveticin_J | Bacteriolysin | FHNXY49-L5 | Bacteriocin_helveticin_J | Bacteriolysin |
|  | Pediocin | ClassIIa |  | bacteriocin_LS2chainb | ClassIId |
| FGSYC19-L1 | bacteriocin_LS2chaina | ClassIId |  | bacteriocin_LS2chaina | ClassIId |
|  | bacteriocin_LS2chainb | ClassIId | FHNXY52-L2 | Pediocin | ClassIIa |
|  | Bacteriocin_helveticin_J | Bacteriolysin |  | Bacteriocin_helveticin_J | Bacteriolysin |
|  | Pediocin | ClassIIa |  | bacteriocin_LS2chaina | ClassIId |
| FGSYC2-L2 | Pediocin | ClassIIa |  | bacteriocin_LS2chainb | ClassIId |
|  | Bacteriocin_helveticin_J | Bacteriolysin | FHNXY54-L2 | Bacteriocin_helveticin_J | Bacteriolysin |
| FGSYC23-L3 | Bacteriocin_helveticin_J | Bacteriolysin |  | bacteriocin_LS2chaina | ClassIId |
|  | Pediocin | ClassIIa |  | bacteriocin_LS2chainb | ClassIId |
| FGSYC34-L2 | Bacteriocin_helveticin_J | Bacteriolysin | FHNXY56-L1 | Pediocin | ClassIIa |
|  | Pediocin | ClassIIa |  | Acidocin_LF221B(GassericinK7B) | ClassIIb |
| FGSYC38-L3 | Pediocin | ClassIIa |  | Gassericin_T | ClassIIb |
|  | Bacteriocin_helveticin_J | Bacteriolysin |  | Bacteriocin_helveticin_J | Bacteriolysin |
|  | bacteriocin_LS2chaina | ClassIId |  | Acidocin_B | ClassIIc |
|  | bacteriocin_LS2chainb | ClassIId |  | Gassericin_A | ClassIIc |
| FGSYC41-L1 | Pediocin | ClassIIa |  | bacteriocin_LS2chaina | ClassIId |
|  | Helveticin-J |  |  | bacteriocin_LS2chainb | ClassIId |
|  | Bacteriocin_helveticin_J | Bacteriolysin | FHNXY6-L2 | Gassericin_T | ClassIIb |
| FGSYC43-L1 | bacteriocin_LS2chainb | ClassIId |  | Acidocin_LF221B(GassericinK7B) | ClassIIb |
|  | bacteriocin_LS2chaina | ClassIId |  | Pediocin | ClassIIa |
|  | Bacteriocin_helveticin_J | Bacteriolysin |  | Bacteriocin_helveticin_J | Bacteriolysin |
|  | Gassericin_T | ClassIIb |  | bacteriocin_LS2chainb | ClassIId |
|  | Acidocin_LF221B(GassericinK7B) | ClassIIb |  | bacteriocin_LS2chaina | ClassIId |
| FGSYC7-L1 | Bacteriocin_helveticin_J | Bacteriolysin |  | Acidocin_B | ClassIIc |
|  | Gassericin_T | ClassIIb | FHNXY61-L1 | Bacteriocin_helveticin_J | Bacteriolysin |
|  | Acidocin_LF221B(GassericinK7B) | ClassIIb |  | bacteriocin_LS2chaina | ClassIId |
|  | Pediocin | ClassIIa |  | bacteriocin_LS2chainb | ClassIId |
|  | bacteriocin_LS2chaina | ClassIId | FHuNCS1-L1 | Acidocin_B | ClassIIc |
|  | bacteriocin_LS2chainb | ClassIId |  | Pediocin | ClassIIa |
| FGSYC79-L2 | Pediocin | ClassIIa |  | Acidocin_LF221B(GassericinK7B) | ClassIIb |
|  | Acidocin_LF221B(GassericinK7B) | ClassIIb |  | Gassericin_T | ClassIIb |
|  | Gassericin_T | ClassIIb |  | bacteriocin_LS2chaina | ClassIId |
|  | Bacteriocin_helveticin_J | Bacteriolysin |  | bacteriocin_LS2chainb | ClassIId |
|  | bacteriocin_LS2chaina | ClassIId |  | Bacteriocin_helveticin_J | Bacteriolysin |
|  | bacteriocin_LS2chainb | ClassIId | FJSCZD2-L1 | bacteriocin_LS2chaina | ClassIId |
| FGSYC9-L1 | Bacteriocin_helveticin_J | Bacteriolysin |  | bacteriocin_LS2chainb | ClassIId |
|  | Gassericin_T | ClassIIb |  | Acidocin_B | ClassIIc |
|  | Acidocin_LF221B(GassericinK7B) | ClassIIb |  | Gassericin_A | ClassIIc |
|  | Pediocin | ClassIIa |  | Pediocin | ClassIIa |
| FGSZY12-L1 | Gassericin_T | ClassIIb |  | Acidocin_LF221B(GassericinK7B) | ClassIIb |
|  | Acidocin_LF221B(GassericinK7B) | ClassIIb |  | Gassericin_T | ClassIIb |
|  | Pediocin | ClassIIa |  | Bacteriocin_helveticin_J | Bacteriolysin |
|  | bacteriocin_LS2chaina | ClassIId | FJSSZ1-L1 | bacteriocin_LS2chaina | ClassIId |
|  | bacteriocin_LS2chainb | ClassIId |  | bacteriocin_LS2chainb | ClassIId |
|  | Bacteriocin_helveticin_J | Bacteriolysin |  | Acidocin_B | ClassIIc |
| FGSZY27-L1 | Pediocin | ClassIIa |  | Gassericin_A | ClassIIc |
|  | Acidocin_LF221B(GassericinK7B) | ClassIIb |  | Pediocin | ClassIIa |
|  | Gassericin_T | ClassIIb |  | Acidocin_LF221B(GassericinK7B) | ClassIIb |
|  | Bacteriocin_helveticin_J | Bacteriolysin |  | Gassericin_T | ClassIIb |
|  | bacteriocin_LS2chaina | ClassIId |  | Bacteriocin_helveticin_J | Bacteriolysin |
|  | bacteriocin_LS2chainb | ClassIId | FJSWX10-L4 | Pediocin | ClassIIa |
| FGSZY29-L8 | Bacteriocin_helveticin_J | Bacteriolysin |  | Acidocin_LF221B(GassericinK7B) | ClassIIb |
|  | Gassericin_T | ClassIIb |  | Gassericin_T | ClassIIb |
|  | Acidocin_LF221B(GassericinK7B) | ClassIIb |  | Acidocin_B | ClassIIc |
|  | Pediocin | ClassIIa |  | Gassericin_A | ClassIIc |
|  | bacteriocin_LS2chaina | ClassIId |  | bacteriocin_LS2chaina | ClassIId |
|  | bacteriocin_LS2chainb | ClassIId |  | bacteriocin_LS2chainb | ClassIId |
|  | Acidocin_B | ClassIIc |  | Bacteriocin_helveticin_J | Bacteriolysin |
| FGSZY30-L1 | Bacteriocin_helveticin_J | Bacteriolysin | FJSWX21-L2 | Bacteriocin_helveticin_J | Bacteriolysin |
|  | Gassericin_T | ClassIIb |  | Gassericin_T | ClassIIb |
|  | Acidocin_LF221B(GassericinK7B) | ClassIIb |  | Acidocin_LF221B(GassericinK7B) | ClassIIb |
|  | Pediocin | ClassIIa |  | Pediocin | ClassIIa |
|  | bacteriocin_LS2chaina | ClassIId |  | bacteriocin_LS2chaina | ClassIId |
|  | bacteriocin_LS2chainb | ClassIId |  | bacteriocin_LS2chainb | ClassIId |
|  | Acidocin_B | ClassIIc | FJSWX33-L2 | Acidocin_B | ClassIIc |
| FGSZY36-L1 | Bacteriocin_helveticin_J | Bacteriolysin |  | bacteriocin_LS2chaina | ClassIId |
|  | Gassericin_T | ClassIIb |  | bacteriocin_LS2chainb | ClassIId |
|  | Acidocin_LF221B(GassericinK7B) | ClassIIb |  | Bacteriocin_helveticin_J | Bacteriolysin |
|  | Pediocin | ClassIIa | FJSWX6-L7 | Pediocin | ClassIIa |
|  | bacteriocin_LS2chaina | ClassIId |  | Helveticin-J |  |
|  | bacteriocin_LS2chainb | ClassIId | FJSWX9-L2 | bacteriocin_LS2chaina | ClassIId |
|  | Acidocin_B | ClassIIc |  | bacteriocin_LS2chainb | ClassIId |
| FHeBCZ3-L3 | Bacteriocin_helveticin_J | Bacteriolysin |  | Acidocin_B | ClassIIc |
|  | bacteriocin_LS2chaina | ClassIId |  | Gassericin_A | ClassIIc |
|  | bacteriocin_LS2chainb | ClassIId |  | Bacteriocin_helveticin_J | Bacteriolysin |
| FHLJDQ3-L5 | Pediocin | ClassIIa |  | Gassericin_T | ClassIIb |
|  | Acidocin_LF221B(GassericinK7B) | ClassIIb |  | Acidocin_LF221B(GassericinK7B) | ClassIIb |
|  | Gassericin_T | ClassIIb |  | Pediocin | ClassIIa |
|  | Bacteriocin_helveticin_J | Bacteriolysin | FJXPY18-L3 | Bacteriocin_helveticin_J | Bacteriolysin |
|  | Acidocin_B | ClassIIc |  | Pediocin | ClassIIa |
|  | bacteriocin_LS2chaina | ClassIId | FJXPY24-L2 | bacteriocin_LS2chaina | ClassIId |
|  | bacteriocin_LS2chainb | ClassIId |  | bacteriocin_LS2chainb | ClassIId |
| FHNFQ10-L1 | Pediocin | ClassIIa |  | Bacteriocin_helveticin_J | Bacteriolysin |
|  | Acidocin_LF221B(GassericinK7B) | ClassIIb | FJXPY26-L4 | Pediocin | ClassIIa |
|  | Gassericin_T | ClassIIb |  | Acidocin_LF221B(GassericinK7B) | ClassIIb |
|  | Bacteriocin_helveticin_J | Bacteriolysin |  | Gassericin_T | ClassIIb |
|  | bacteriocin_LS2chainb | ClassIId |  | Acidocin_B | ClassIIc |
|  | bacteriocin_LS2chaina | ClassIIa |  | bacteriocin_LS2chainb | ClassIId |
| FHNFQ11-L7 | Pediocin | ClassIIb |  | bacteriocin_LS2chaina | ClassIId |
|  | Acidocin_LF221B(GassericinK7B) | ClassIIb |  | Bacteriocin_helveticin_J | Bacteriolysin |
|  | Gassericin_T | ClassIId | FNMGHHHT1-L5 | Gassericin_A | ClassIIc |
|  | bacteriocin_LS2chaina | ClassIId |  | Acidocin_B | ClassIIc |
|  | bacteriocin_LS2chainb | Bacteriolysin |  | Pediocin | ClassIIa |
|  | Bacteriocin_helveticin_J | ClassIId |  | bacteriocin_LS2chaina | ClassIId |
| FHNFQ14-L5 | bacteriocin_LS2chaina | ClassIId |  | bacteriocin_LS2chainb | ClassIId |
|  | bacteriocin_LS2chainb | Bacteriolysin |  | Gassericin_T | ClassIIb |
|  | Bacteriocin_helveticin_J | ClassIId |  | Acidocin_LF221B(GassericinK7B) | ClassIIb |
| FHNFQ15-L4 | bacteriocin_LS2chaina | ClassIId |  | Bacteriocin_helveticin_J | Bacteriolysin |
|  | bacteriocin_LS2chainb | Bacteriolysin | FNMGHLBE17-L3 | bacteriocin_LS2chainb | ClassIId |
|  | Bacteriocin_helveticin_J | Bacteriolysin |  | bacteriocin_LS2chaina | ClassIId |
| FHNFQ16-L5 | Bacteriocin_helveticin_J | ClassIIb |  | Acidocin_B | ClassIIc |
|  | Gassericin_T | ClassIIb |  | Gassericin_A | ClassIIc |
|  | Acidocin_LF221B(GassericinK7B) | ClassIIa |  | Pediocin | ClassIIa |
|  | Pediocin | ClassIId |  | Acidocin_LF221B(GassericinK7B) | ClassIIb |
|  | bacteriocin_LS2chaina | ClassIId |  | Gassericin_T | ClassIIb |
|  | bacteriocin_LS2chainb | ClassIIc |  | Bacteriocin_helveticin_J | Bacteriolysin |
|  | Acidocin_B | ClassIId | FNMGHLBE20-L5 | Pediocin | ClassIIa |
| FHNFQ20-L1 | bacteriocin_LS2chaina | ClassIId |  | Bacteriocin_helveticin_J | Bacteriolysin |
|  | bacteriocin_LS2chainb | ClassIIa |  | bacteriocin_LS2chainb | ClassIId |
|  | Pediocin | Bacteriolysin |  | bacteriocin_LS2chaina | ClassIId |
|  | Bacteriocin_helveticin_J | ClassIIa | FSDHZ21-L1 | Bacteriocin_helveticin_J | Bacteriolysin |
| FHNFQ25-L3 | Pediocin | ClassIIb |  | Pediocin | ClassIIa |
|  | Acidocin_LF221B(GassericinK7B) | ClassIIb | FSDHZD3-L5 | Pediocin | ClassIIa |
|  | Gassericin_T | ClassIIc |  | Acidocin_LF221B(GassericinK7B) | ClassIIb |
|  | Acidocin_B | ClassIId |  | Gassericin_T | ClassIIb |
|  | bacteriocin_LS2chaina | ClassIId |  | Acidocin_B | ClassIIc |
|  | bacteriocin_LS2chainb | Bacteriolysin |  | Gassericin_A | ClassIIc |
|  | Bacteriocin_helveticin_J | Bacteriolysin |  | bacteriocin_LS2chaina | ClassIId |
| FHNFQ28-L4 | Bacteriocin_helveticin_J | ClassIIa |  | bacteriocin_LS2chainb | ClassIId |
|  | Pediocin | ClassIId |  | Bacteriocin_helveticin_J | Bacteriolysin |
|  | bacteriocin_LS2chaina | ClassIId | FSDYT1-L1 | bacteriocin_LS2chainb | ClassIId |
|  | bacteriocin_LS2chainb | ClassIId |  | bacteriocin_LS2chaina | ClassIId |
| FHNFQ29-L2 | bacteriocin_LS2chaina | ClassIId |  | Bacteriocin_helveticin_J | Bacteriolysin |
|  | bacteriocin_LS2chainb | Bacteriolysin |  | Pediocin | ClassIIa |
|  | Bacteriocin_helveticin_J | ClassIIa | FTJWQ2-L9 | Bacteriocin_helveticin_J | Bacteriolysin |
|  | Pediocin | ClassIId |  | Pediocin | ClassIIa |
|  | Enterolysin_A | ClassIIa | FZJHZD1-M5 | Bacteriocin_helveticin_J | Bacteriolysin |
| FHNFQ3-L8 | Pediocin | ClassIIb |  | Pediocin | ClassIIa |
|  | Acidocin_LF221B(GassericinK7B) | ClassIIb | M2CF21-L1 | bacteriocin_LS2chaina | ClassIId |
|  | Gassericin_T | ClassIIc |  | bacteriocin_LS2chainb | ClassIId |
|  | Acidocin_B | ClassIIc |  | Bacteriocin_helveticin_J | Bacteriolysin |
|  | Gassericin_A | ClassIId |  | Pediocin | ClassIIa |
|  | bacteriocin_LS2chaina | ClassIId | FGSYC8-L2 | Bacteriocin_helveticin_J | Bacteriolysin |
|  | bacteriocin_LS2chainb | Bacteriolysin | FHeNJZ11-L9 | Bacteriocin_helveticin_J | Bacteriolysin |
|  | Bacteriocin_helveticin_J | ClassIId | FHNFQ56-L1 | Bacteriocin_helveticin_J | Bacteriolysin |
| FHNFQ34-L1 | bacteriocin_LS2chaina | ClassIId | FHNFQ57-L4 | Bacteriocin_helveticin_J | Bacteriolysin |
|  | bacteriocin_LS2chainb | Bacteriolysin |  | Pediocin | ClassIIa |
|  | Bacteriocin_helveticin_J | Bacteriolysin | FHNFQ60-L1 | Bacteriocin_helveticin_J | Bacteriolysin |
| FJSWX21-L2 | Bacteriocin_helveticin_J | Bacteriolysin | FHNXY58-L2 | Bacteriocin_helveticin_J | Bacteriolysin |
|  | Acidocin_LF221B(GassericinK7B) | ClassIIb | FHNXY9-L1 | Bacteriocin_helveticin_J | Bacteriolysin |
|  | Gassericin_T | ClassIIc | FJXPY34-L1 | Bacteriocin_helveticin_J | Bacteriolysin |
|  | Pediocin | ClassIId | FJXPY37-L3 | Bacteriocin_helveticin_J | Bacteriolysin |
|  | bacteriocin_LS2chaina | ClassIId | FJXPY5-L2 | Bacteriocin_helveticin_J | Bacteriolysin |
|  | bacteriocin_LS2chainb | ClassIId | FJXPY6-L1 | Bacteriocin_helveticin_J | Bacteriolysin |
| FHNXY12-L2 | Pediocin | ClassIId | FNMGHLBE6-L1 | Bacteriocin_helveticin_J | Bacteriolysin |
|  | Acidocin_LF221B(GassericinK7B) | ClassIIb | FSDHZ19-L1 | Bacteriocin_helveticin_J | Bacteriolysin |
|  | Gassericin_T | ClassIIc |  |  |  |
|  | Bacteriocin_helveticin_J | Bacteriolysin |  |  |  |
|  | bacteriocin_LS2chaina | ClassIId |  |  |  |
|  | bacteriocin_LS2chainb | ClassIId |  |  |  |
