# Supplementary figures and images for: Comparative analysis of Lactobacillus gasseri from Chinese subjects reveals a new species-level taxa
Source: BMC Genomics. 2020 Feb 3;21:119. doi: 10.1186/s12864-020-6527-y (PMC6998098; doi:10.1186/s12864-020-6527-y)

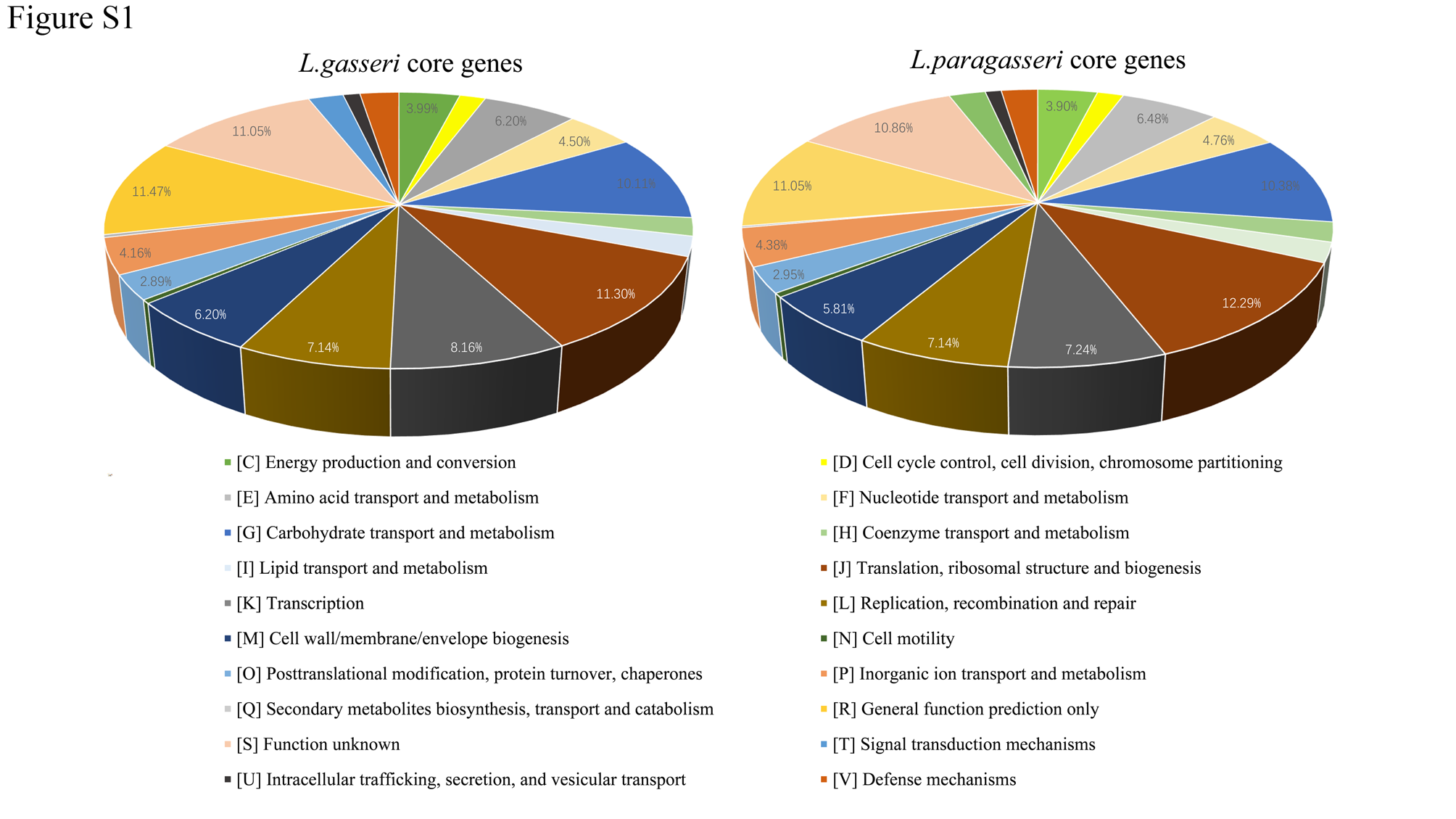

Supplement: Supplementary file 5 — Additional file5: Figure S1. Functional assignment of the L. paragassei (a) and L. gasseri core genome based on the COG database [file 12864_2020_6527_MOESM5_ESM.tif]
